# ModelistsGCN: a multimodal graph convolutional network framework for single-cell spatial transcriptomic cell typing

**DOI:** 10.1093/bib/bbag340

**Published:** 2026-06-22

**Authors:** Noa Konforti, Tal Goldberg, Michal Danino-Levi, Yael Ilan, Shahar Alon

**Affiliations:** The Alexander Kofkin Faculty of Engineering, Bar-Ilan University, Ramat Gan 5290002, Israel; Institute for Nanotechnology and Advanced Materials (BINA), Bar-Ilan University, Ramat Gan 5290002, Israel; The Gonda Multidisciplinary Brain Research Center, Bar-Ilan University, Ramat Gan 5290002, Israel; The Alexander Kofkin Faculty of Engineering, Bar-Ilan University, Ramat Gan 5290002, Israel; Institute for Nanotechnology and Advanced Materials (BINA), Bar-Ilan University, Ramat Gan 5290002, Israel; The Gonda Multidisciplinary Brain Research Center, Bar-Ilan University, Ramat Gan 5290002, Israel; The Alexander Kofkin Faculty of Engineering, Bar-Ilan University, Ramat Gan 5290002, Israel; Institute for Nanotechnology and Advanced Materials (BINA), Bar-Ilan University, Ramat Gan 5290002, Israel; The Gonda Multidisciplinary Brain Research Center, Bar-Ilan University, Ramat Gan 5290002, Israel; The Alexander Kofkin Faculty of Engineering, Bar-Ilan University, Ramat Gan 5290002, Israel; Institute for Nanotechnology and Advanced Materials (BINA), Bar-Ilan University, Ramat Gan 5290002, Israel; The Gonda Multidisciplinary Brain Research Center, Bar-Ilan University, Ramat Gan 5290002, Israel; The Alexander Kofkin Faculty of Engineering, Bar-Ilan University, Ramat Gan 5290002, Israel; Institute for Nanotechnology and Advanced Materials (BINA), Bar-Ilan University, Ramat Gan 5290002, Israel; The Gonda Multidisciplinary Brain Research Center, Bar-Ilan University, Ramat Gan 5290002, Israel

**Keywords:** single-cell spatial transcriptomics, cell type identification, graph convolutional network, semi-supervised learning, multimodal data integration

## Abstract

Spatial transcriptomics technologies currently face a trade-off between spatial and molecular resolution. High-resolution single-cell methods such as MERFISH and Expansion Sequencing (ExSeq) resolve individual cells but typically profile limited gene panels, restricting the direct application of transcriptome-based cell typing strategies developed for single-cell RNA sequencing. Consequently, accurate cell-type identification in spatial single-cell data remains challenging when transcriptomic coverage per cell is sparse. Here, we introduce ModelistsGCN, a semi-supervised multimodal graph convolutional framework that integrates gene expression, spatial proximity, and quantitative cellular morphology for spatial single-cell cell typing. ModelistsGCN uses a small set of high-confidence representative cells, defined by marker-gene enrichment, to guide clustering while preserving flexibility in identifying cell types. By incorporating spatial neighborhood structure and interpretable morphological features alongside gene expression, the method compensates for limited molecular coverage and strengthens cell-type discrimination. Across a well-annotated ExSeq mouse visual cortex dataset and metastatic breast cancer tissues profiled by MERFISH and ExSeq, ModelistsGCN consistently demonstrated higher agreement with reference annotations, improved cluster separation, and stronger marker-gene coherence compared to existing spatial clustering approaches. ModelistsGCN improves cell-type inference in single-cell spatial transcriptomic datasets with limited gene coverage.

## Graphical Abstract

**Graphical Abstract:**
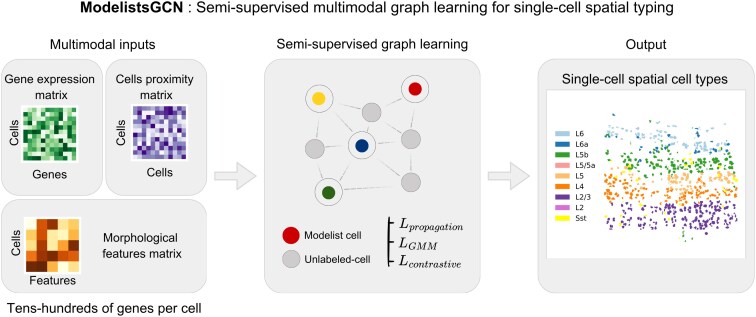

## Introduction

Spatial transcriptomics technologies currently face a trade-off between spatial and molecular resolution. Methods providing high spatial precision, capable of resolving individual cells or even subcellular structures, typically profile a limited number of genes, whereas transcriptome-wide approaches often lack the spatial accuracy required to assign transcripts to individual cells [1]. For example, RNA-capture approaches such as Spatial Transcriptomics (ST) [2] and Slide-seq [3] enable near-whole-transcriptome coverage but are constrained by spot size, limiting single-cell characterization. In contrast, in situ fluorescence-based methods achieve single-cell or subcellular resolution yet measure only a subset of expressed genes. These methods include MERFISH [4], its commercial implementation MERSCOPE (Vizgen), Xenium (10× Genomics) [5], SeqFISH, STARmap, ISS, PRISM and related techniques [6–11], as well as targeted Expansion Sequencing (ExSeq) [12], a super-resolution in situ sequencing technology developed by us and others.

This trade-off complicates spatial cell typing, which is essential for studying tissue organization and local cell–cell interactions. Most existing molecular cell typing frameworks were largely developed for conventional single cell RNA sequencing [13], which captures near-complete transcriptomes from dissociated cells. In contrast, spatial single-cell measurements typically provide sparse transcriptomic coverage, limiting the direct transfer of transcriptome-based cell typing strategies to spatial data. Consequently, gene expression-based cell typing alone may be insufficient in spatial single-cell data, as key marker genes may be undetected and reduced feature dimensionality weakens conventional clustering performance.

Cell type annotation of spatial single-cell data can be achieved by mapping spatial expression profiles to reference atlases derived from single cell RNA sequencing. While this enables cell type annotation using transcriptome-wide references, differences in measurement modality and transcriptomic coverage limit the reliability of atlas-based cell type transfer [14]. An extension of this approach is to perform two complementary experiments on consecutive tissue sections, one generating single-cell spatial data and the other standard single cell RNA sequencing. In this setting, cell types are defined in the expression space of the single cell RNA sequencing data, and cells from the spatial dataset are then projected into this space for annotation [5]. However, mapping spatial data with limited gene coverage onto genome-wide reference spaces remains challenging and can reduce annotation reliability.

Despite the rich contextual information provided by spatial data, including precise cellular positioning and morphological features, until recently spatial cell typing approaches relied primarily on gene expression, and the limited incorporation of spatial cues has not consistently improved classification accuracy [15–17]. Several recent algorithms, such as SpaGCN and GraphST [18, 19], have introduced graph-based models to incorporate spatial information into cell typing. However, these frameworks are tailored to spot-based platforms like Visium (10× Genomics) [2], which lack single-cell resolution and detailed morphological features. For instance, SpaGCN integrates features derived from histological RGB images rather than explicit single-cell morphological descriptors, while GraphST was developed originally for genome-wide expression profiles rarely available in single-cell spatial protocols. Consequently, both methods might perform suboptimally when applied to single-cell spatial data [16]. Other tools, including Squidpy [20], provide flexible frameworks for analyzing spatial data and allow limited integration of image-derived features. Yet these approaches typically rely on 2D histological images, such as H&E staining, which do not allow direct extraction of single-cell 3D morphological features.

Thus, while several computational methods for spatial cell typing have been developed, accurate single-cell spatial cell typing remains an active methodological challenge. Accordingly, Zhang *et al*. [14] evaluated six different methods for assigning cell types to the same single-cell spatial datasets and reported substantial variability in cell type assignments across methods. A focused comparison of existing spatial cell typing methods, highlighting limitations addressed in this study, is provided in Table S1. Importantly, existing frameworks often conflate spot-level domain segmentation with true single-cell classification, leading to ambiguity and reduced interpretability at the single-cell level. Specifically, two key limitations persist: (i) insufficient integration of interpretable single-cell morphological features, particularly in three dimensions, and (ii) limited robustness to partial transcriptomic coverage per cell, where tens to hundreds of genes are measured rather than near-complete transcriptomes.

Among the compared methods (Table S1), only STELLAR [21], CCST [22], and BASS [23] were explicitly designed for single-cell spatial cell typing. BASS performs joint single-cell clustering together with spatial domain detection and is designed to model multiple tissue sections or samples simultaneously, rather than focusing on cell-type assignment within individual samples alone. CCST represents a fully unsupervised approach, performing spatial clustering based on gene expression and spatial graphs, and was evaluated on datasets with near genome-wide gene coverage. STELLAR, in contrast, is a reference-based method that transfers cell-type annotations from annotated datasets.

Here, we introduce ModelistsGCN, a graph convolutional framework that directly addresses the challenges of limited transcriptomic depth and the lack of interpretable morphological integration. Compared to CCST and STELLAR, ModelistsGCN occupies an intermediate position by utilizing limited prior knowledge to support cell-type assignment under constrained gene coverage. By jointly leveraging molecular, spatial, and morphological information, ModelistsGCN improves cell-type inference in spatial single-cell datasets with sparse transcriptomic profiling.

## Methods

### Methods summary

This section outlines the overall design and conceptual workflow of ModelistsGCN, while specific implementation details, mathematical formulations, and sensitivity analyses are provided in the Supplementary Methods Section S1.

**Figure 1:**
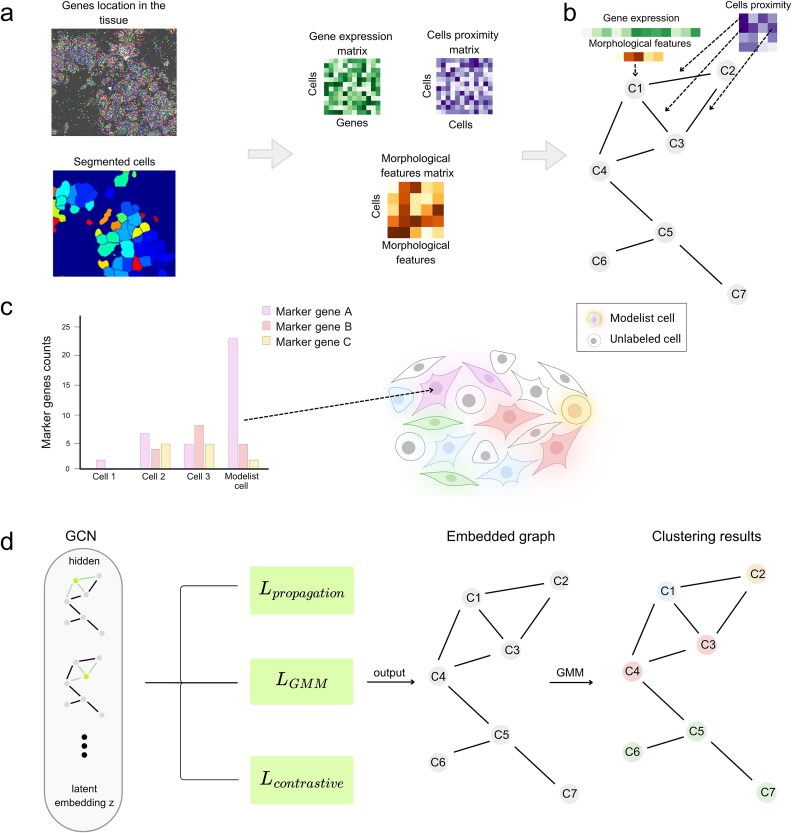
Overview of the ModelistsGCN method. (a) Starting from single-cell spatial transcriptomic data and segmented cell images, we use gene expression matrices together with two additional complementary data modalities: morphological feature matrices extracted from cell segmentation and cell–cell proximity matrices computed from spatial coordinates. (b) Graph construction integrates gene expression, morphology, and spatial proximity by representing cells as nodes connected through adjacency relationships, forming a multimodal graph. (c) A subset of cells enriched for marker-gene expression is defined as modelist cells, which provide semi-supervised guidance for clustering. Created in BioRender. Konforti, N. (2026) https://BioRender.com/fbmou94. (d) The ModelistsGCN architecture processes this graph through graph convolutional layers and jointly optimizes multiple objectives, including propagation loss, GMM pull loss, and contrastive loss, to generate an embedded graph. A GMM applied on the embeddings produces clustering results, yielding cell type classification.

**Figure 2:**
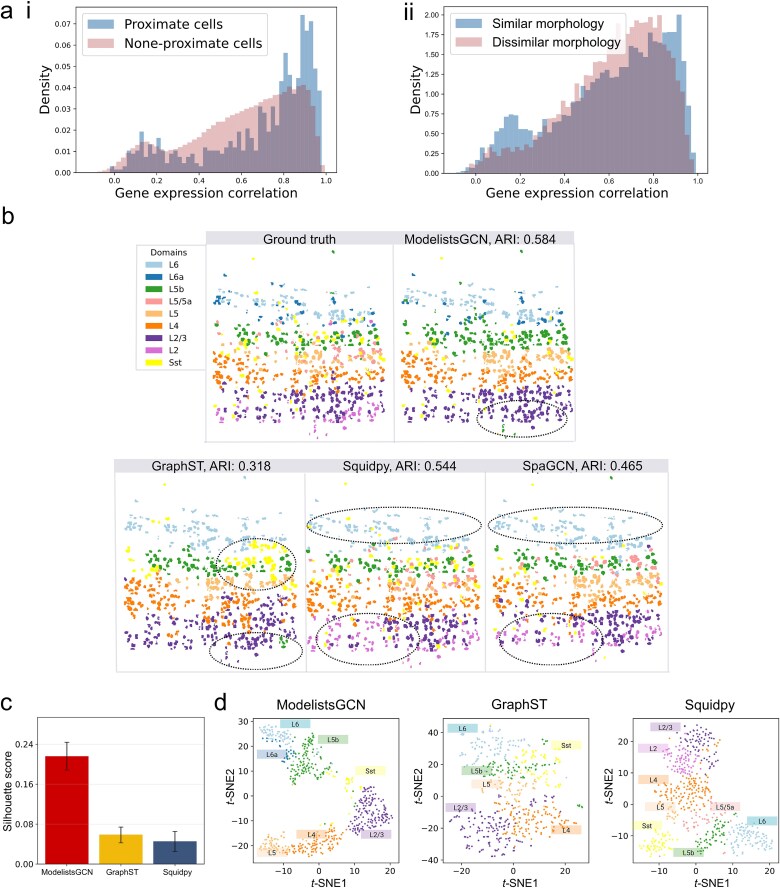
Mouse visual cortex dataset performance. (a) (i) Distribution of pairwise gene expression correlations shows that proximate cells (<10 μm apart) exhibit significantly higher similarity than non-proximate cells (permutation and KS test, *P* = 1.55 × 10^−27^). (ii) Same analysis as in (i), but comparing morphologically similar and dissimilar cell pairs. Morphological similarity was defined in the space of extracted morphological features using the first principal component, with the top and bottom 5% of pairwise distances classified as similar and dissimilar, respectively. Morphologically similar cells exhibit higher gene-expression correlation (KS test, *P* = 1.11 × 10^−13^). (b) Spatial mapping of cell clusters reveals the characteristic layered distribution of cell types within the visual cortex. Each cell is colored by its assigned cluster, and ARI scores quantify the agreement of the different methods with the ground truth annotations. ModelistsGCN achieves the highest ARI (0.584), outperforming GraphST (0.318) Squidpy (0.544) and SpaGCN (0.465). Black dotted outlines indicate regions where cell-type assignment is visually imperfect. (c) Mean silhouette scores computed on the embedding space; error bars indicate the standard error of the mean (STE) estimated via a realization test. ModelistsGCN achieves higher cluster separation compared to GraphST and Squidpy. (d) Two-dimensional *t*-SNE projections of the learned embeddings for each method, colored by predicted cell types, showing better-separated clusters for ModelistsGCN, whereas GraphST and Squidpy exhibit increased overlap between cell populations. SpaGCN is not shown in panels c and d because a stable latent embedding could not be obtained for low-gene-number data.

ModelistsGCN was designed for single-cell spatial datasets (Table S1). ModelistsGCN combines a Graph Convolutional Network (GCN) [24] with a Gaussian Mixture Model (GMM) to infer cell types by clustering cells based on integrated molecular, spatial, and morphological information (Fig. 1). Starting from two primary inputs, a gene expression matrix (cells × gene counts) and a segmented tissue image defining individual cell boundaries, we derive two complementary data representations. First, a morphological feature matrix is extracted from cell morphology using quantitative descriptors established in prior literature [25–27], including features such as volume, elongation, and sphericity; second, a cell neighborhood matrix is constructed to define a spatial proximity graph (Fig. 1a and b). In this graph, each cell is represented as a node, and edges connect neighboring cells whose minimum inter-cell distance is below 10 μm, reflecting a typical cell-scale neighborhood. At this distance threshold, transcriptional similarity between proximate cells was consistently higher than between more distant cells (Fig. 2a and Fig. S1). Node features are defined by concatenating gene expression profiles and morphological descriptors, both z-scored prior to integration.

In tissue profiling experiments, broad cell types are often anticipated based on prior biological knowledge; accordingly, ModelistsGCN is designed for datasets in which a small number of such cell types are expected (Table S2), while single-cell annotations are unavailable. Importantly, prior biological knowledge is used to guide ModelistsGCN rather than constrain it, allowing the discovery of additional cell types or states (Table S2). To incorporate this prior biological knowledge, ModelistsGCN identifies a subset of modelist cells, defined as high-confidence representatives of expected cell types selected based on marker-gene enrichment (Fig. 1c and Fig. S2). These modelist cells are used to initialize the GMM in the latent embedding space, anchoring the representation around biologically meaningful cell-type modes (Fig. S3).

The GCN encoder receives gene expression and morphology as node features and propagates information across the tissue graph, producing latent embeddings for all cells that reflect both intrinsic cellular profiles and local neighborhood context (Fig. 1d). Unlike standard post-hoc clustering, the GMM is embedded within the learning objective and trained jointly with the encoder, allowing the latent embedding and cluster boundaries to co-evolve during training. Final cell-type assignments are obtained from the learned GMM components in this embedding space (Fig. 1d).

## Results

### ModelistsGCN overview

ModelistsGCN is a semi-supervised cell-typing framework that combines a GCN encoder with a GMM to cluster spatial single cells (Fig. 1 and additional analyses in Fig. S1). In many tissue profiling settings, broad cell categories can be anticipated from prior biological knowledge, even though single-cell annotations are unavailable. ModelistsGCN is designed for such scenarios, where a limited number of expected cell types is known in advance.

The method first identifies a subset of high-confidence representative cells, termed modelist cells, based on marker-gene enrichment for the expected cell types (Fig. 1c and Fig. S2). These cells act as biological anchors that guide clustering (Fig. S3), while still permitting the identification of additional cell types beyond those initially specified (Table S2).

Cells are represented as nodes in a spatial graph constructed from pairwise distances, with edges connecting neighboring cells within a defined micrometer-scale threshold (Fig. 1a and b). Node attributes combine gene expression profiles with quantitative morphological descriptors extracted from segmented cells, including volume and shape-related features.

The GCN processes this multimodal graph to generate a latent embedding for each cell (Fig. 1d). During training, propagation, GMM, and contrastive objectives are jointly optimized to shape the embedding space (Methods). A GMM applied to the learned embeddings produces final cluster assignments. Modelist cells guide the assignment of neighboring cells through controlled label propagation across the graph, with tunable parameters governing both the strength and spatial extent of this propagation (Figs S4–S5). This design allows prior biological knowledge to guide clustering while maintaining flexibility to capture additional structure present in the tissue.

### ModelistsGCN improves cell typing and embedding quality in a well-annotated mouse visual cortex dataset

To evaluate the extent to which non-transcriptomic features enhance cell type identification in single-cell spatial transcriptomics, we analysed an ExSeq dataset with curated cell-type annotations (ground-truth; see Supplementary Methods S1) from the mouse visual cortex [12, 14]. Notably, this dataset contains expression measurements for only 42 genes, a setting typical of single-cell spatial assays (e.g. [11]). We first asked whether spatial proximity between cells indeed provides an informative signal for cell typing. Proximal cells exhibited significantly higher gene expression similarity than distant cells (Fig. 2a). This association disappeared when cell positions were randomly shuffled, confirming that the observed proximity-expression relationship reflects true biological organization (permutation analysis and Kolmogorov–Smirnov (KS) test, *P* = 1.55 × 10^−27^) (Fig. 2a and additional analyses in Fig. S1a). We next asked whether the extracted quantitative morphological features likewise capture information relevant to cell typing. Similarly, cells that were more similar in the extracted morphological feature space exhibited significantly higher transcriptional similarity than morphologically dissimilar cells (KS test, *P* = 1.11 × 10^−13^; Fig. 2a and parameter sensitivity tests in Fig. S1b).

When applied to the ExSeq mouse visual cortex dataset, ModelistsGCN achieved a mean Adjusted Rand Index (ARI) [28] score of 0.584, where ARI = 1.0 indicates perfect agreement with ground-truth labels and 0.0 corresponds to random assignment (Fig. 2b). Notably, ModelistsGCN outperformed three other spatial clustering methods evaluated on the same dataset, namely the graph-based approaches GraphST (ARI = 0.318), Squidpy (ARI = 0.544), and SpaGCN (ARI = 0.465). Although some mixing between cortical layers was observed across all methods, including ModelistsGCN, the extent of layer mixing was more pronounced in the alternative approaches (Fig. 2b).

In addition to improved label agreement, ModelistsGCN yielded improved cluster separation. This was reflected by the highest mean silhouette [29] score computed in the learned embedding space, indicating improved within-cluster cohesion and between-cluster separation relative to competing methods (Fig. 2c). Visualization of this same embedding further revealed clear separation between upper (layers 2–4) and deeper (layers 5–6) cortical populations, whereas other methods showed increased overlap and less well-defined layer-specific boundaries (Fig. 2d).

### ModelistsGCN improves clustering in metastatic breast cancer tissues

We next consider the more typical setting in which ground-truth cell-type annotations are unavailable. In this setting, ModelistsGCN identifies a small set of modelist cells defined by marker-gene enrichment (Table S3), which anchor representation learning and enable biologically grounded clustering in unlabeled tissues. Specifically, we applied ModelistsGCN together with the same three established and commonly used graph-based methods, SpaGCN, GraphST, and Squidpy, to five ExSeq metastatic breast cancer tissues profiled with a 300-gene panel [30]. In the absence of ground-truth labels, we evaluated each method using three complementary criteria (Methods): (i) agreement between inferred labels and expected cell types for modelist cells, quantified by ARI; (ii) clustering quality, assessed by the silhouette score in the learned embedding space; and (iii) biological coherence of inferred clusters, evaluated by enrichment of known cell-type marker genes.

**Figure 3:**
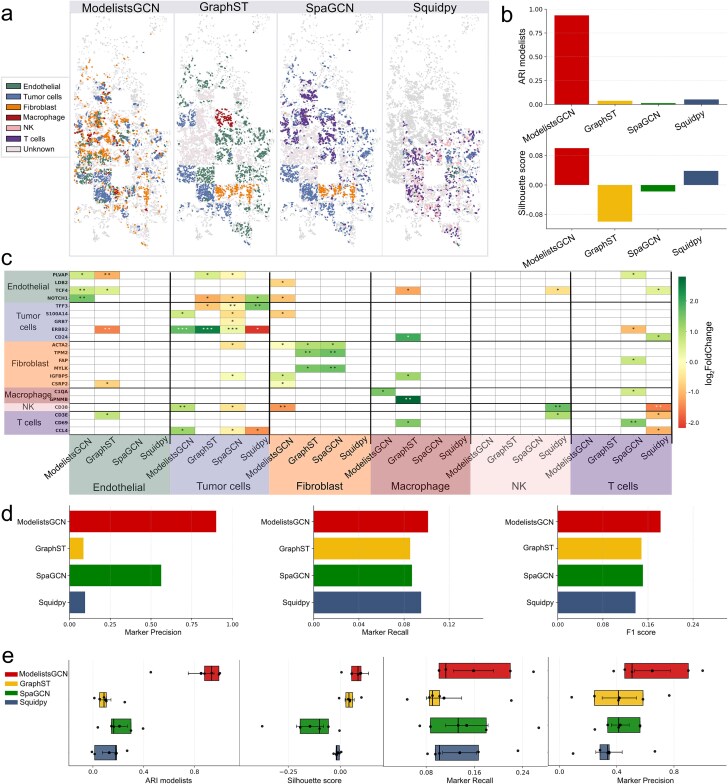
Comparative evaluation of cell-type inference performance across methods on ExSeq metastatic breast cancer data. (a) Spatial clustering results for a representative tissue section (labeled ‘ExSeq-982’), colored by predicted cell types for ModelistsGCN, GraphST, SpaGCN, and Squidpy. Gray points indicate cells assigned to clusters without enrichment of known cell-type markers. (b) Quantitative evaluation on the same representative tissue, showing the ARI (top) and mean silhouette score computed in the embedding space (bottom). (c) Heatmap showing differential expression of cell-type marker genes in the representative tissue (methods). Green indicates upregulated markers and red indicates downregulated markers. Adjusted *P*-values (*q*) are indicated as follows: ^*^q < 10^−3^; ^**^q < 10^−12^; ^***^q < 10^−30^. Note that natural killer (NK) cells were not identified in this sample by ModelistsGCN, GraphST, or SpaGCN, and T cells were not identified by ModelistsGCN or GraphST. Endothelial cells and macrophages were not identified by SpaGCN or Squidpy, while Squidpy also didn’t identify fibroblasts. (d) Marker-based performance on the representative tissue, showing marker precision, recall, and F1 score for each method. (e) Summary statistics across five ExSeq metastatic breast cancer tissues, shown as boxplots for modelist ARI, silhouette score, marker recall, and marker precision across methods.

In a representative metastatic tissue (Fig. 3a and additional samples in Fig. S6), GraphST and SpaGCN produced sharply separated clusters dominated by individual cell types occupying distinct spatial regions, whereas ModelistsGCN yielded a more interleaved organization with multiple cell types co-occurring across local neighborhoods. At the same time, ModelistsGCN maintained higher agreement between inferred labels and expected cell types for modelist cells and produced embeddings with improved cluster structure (Fig. 3b). Importantly, ModelistsGCN also produced clusters with stronger biological specificity, with marker genes characteristic of endothelial, tumor, and fibroblast populations showing more coherent enrichment within their corresponding clusters compared to competing approaches (Fig. 3c). This biological consistency translated into improved marker-based performance, with higher marker recall and F1 scores in the representative tissue (Fig. 3d).

We next examined whether these trends were consistent across the five metastatic ExSeq tissues. Across all tissues, ModelistsGCN showed strong performance, with higher agreement between inferred labels and expected cell types for modelist cells (ARI), improved silhouette scores, and enhanced marker-based metrics relative to GraphST, SpaGCN, and Squidpy (Fig. 3e). Together, these results show that ModelistsGCN supports effective cell-type clustering in unlabeled metastatic tissues.

### Cross-platform benchmarking using MERFISH datasets

To assess the generalizability of our method across spatial transcriptomic platforms, we evaluated the performance of ModelistsGCN on MERFISH single-cell spatial datasets [30]. We analysed ten metastatic breast cancer tissues profiled using the same 300-gene panel as in the ExSeq experiments (Methods). ModelistsGCN was evaluated alongside SpaGCN, GraphST, and Squidpy using the same label agreement, clustering quality, and marker-based criteria described above.

**Figure 4:**
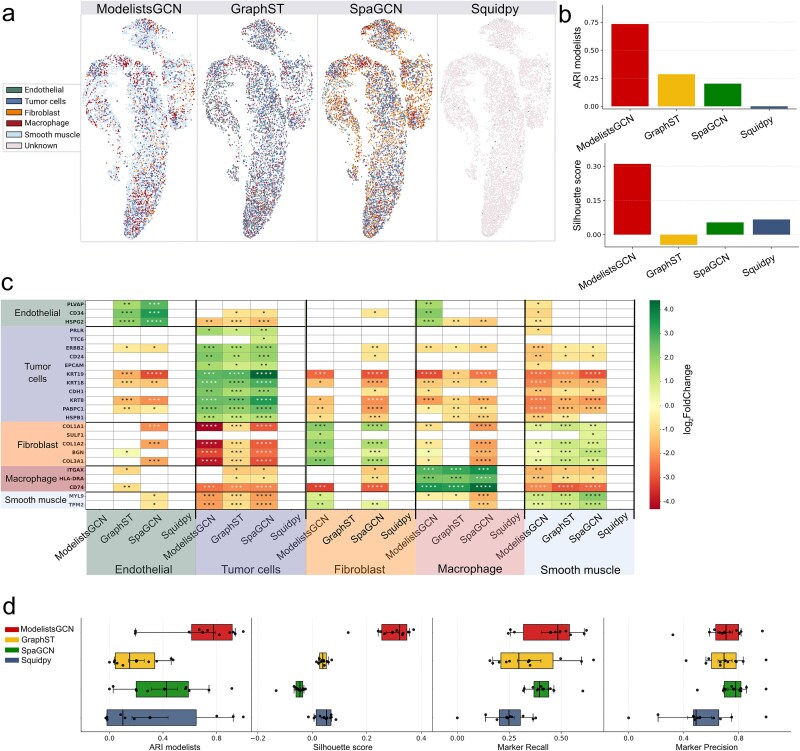
Comparative evaluation of cell-type assignments across methods on MERFISH metastatic breast cancer datasets. (a) Spatial clustering outputs for a representative tissue section (labeled ‘MERFISH-878’), colored by predicted cell types, comparing ModelistsGCN, GraphST, SpaGCN, and Squidpy. Gray points indicate clusters lacking enrichment for known cell-type marker genes. (b) Quantitative evaluation on the same representative tissue, including ARI (top) and mean silhouette score (bottom). (c) Heatmap of differential expression analysis for cell-type-specific marker genes in a single tissue. Green indicates upregulated genes and red indicates downregulated genes. Significance is denoted as follows: ^*^q < 10^−3^; ^**^q < 10^−12^; ^***^q < 10^−30^, ^****^q < 10^−100^. Note that endothelial cells were not identified in this sample by ModelistsGCN, and fibroblast cells were not identified by GraphST. In this tissue, Squidpy did not identify any cell types. (d) Summary statistics across ten MERFISH metastatic breast cancer tissues, shown as boxplots for ARI, silhouette score, marker recall, and marker precision.

In a representative MERFISH tissue (Fig. 4a and additional samples in Fig. S7), despite the highly mixed architecture typical of metastatic tissues, ModelistsGCN achieved the highest agreement with modelist annotations and the strongest clustering structure, reflected by increased ARI and silhouette score relative to GraphST, SpaGCN, and Squidpy (Fig. 4b). Consistent with its improved clustering quality, ModelistsGCN showed clearer enrichment of canonical marker genes within inferred cell-type clusters, including tumor cells, macrophage, smooth muscle, and fibroblast populations, compared to competing methods (Fig. 4c). Finally, statistical evaluation across all ten MERFISH tissues demonstrated that the improved performance of ModelistsGCN was consistent across datasets, with higher ARI, silhouette scores, and marker-based metrics compared to the baseline methods (Fig. 4d). Together, these results indicate that ModelistsGCN generalizes across spatial transcriptomic platforms and performs robustly in complex metastatic microenvironments with highly intermixed cell populations.

We next examined the runtime and memory usage of ModelistsGCN versus other graph-based methods, all using default settings (Fig. S8 and Table S4). Memory usage was similar across methods for ~3000 cells. For ~10 000 cells, ModelistsGCN required ~5 GB RAM, comparable to GraphST and up to 2× higher than SpaGCN and Squidpy. In terms of runtime, ModelistsGCN was approximately 30%–40% faster than Squidpy and SpaGCN, and over 80% faster than GraphST across MERFISH tissues.

### Comparison to additional spatial single-cell methods

We expanded benchmarking across MERFISH breast cancer tissues to include STELLAR, CCST, and BASS, which represent recently developed methods for single-cell spatial transcriptomic cell typing (Table S5). STELLAR is a reference-based method that relies on a spatially annotated reference dataset. We evaluated STELLAR using MERFISH data from one biopsy (MERFISH 514) as a reference and additionally tested whether performance improves when using a reference derived from a different section of the same patient biopsy; however, this did not improve performance, likely due to spatial heterogeneity between non-adjacent sections (~75 μm apart). In contrast, ModelistsGCN does not require an external reference dataset, such as spatial cell-type annotations from a closely matched tissue or adjacent section, which may not be available in practice. Under these conditions, ModelistsGCN consistently outperforms STELLAR across most datasets, with substantial gains in ARI (for example, 0.734 versus 0.252 in MERFISH 878 and 0.899 versus 0.137 in MERFISH 880), alongside improvements in Silhouette score and marker-based metrics.

Compared to CCST, ModelistsGCN consistently achieves higher ARI and Silhouette scores across datasets, with large margins. Marker recall is also consistently improved, while marker precision remains comparable overall. Relative to BASS, ModelistsGCN achieves higher ARI in 6/9 datasets (average improvement approximately 0.2–0.25), with comparable Silhouette scores and a tradeoff between higher recall (ModelistsGCN) and higher precision (BASS). Importantly, ModelistsGCN is substantially more efficient, with runtimes reduced by more than two orders of magnitude compared to CCST and BASS (for example, ~10 seconds versus ~1 hour for MERFISH 880 and < 1 minute versus ~5 hours for MERFISH 944; Table S5).

### Generalization across datasets and impact of morphology

To assess generalizability, we applied ModelistsGCN to a Xenium normal human kidney dataset, which differs from the primary MERFISH and ExSeq breast cancer datasets in both tissue type and technology. As shown in Fig. S9, ModelistsGCN achieves higher Silhouette score, marker recall, and marker precision compared to SpaGCN, which was selected as a representative baseline due to its strong performance in our benchmarks (Figs 3–4), demonstrating consistent performance across distinct biological contexts and platforms.

We further evaluated the contribution of morphological features through a direct comparison of segmentation strategies on the same tissue. In this analysis, membrane-based segmentation, which more accurately delineates cell boundaries than standard DAPI-based approaches, produces improved downstream performance, as reflected by stronger marker-gene enrichment and higher marker recall and precision (Fig. S10). Together, these results support that improved capture of cell morphology, particularly in 3D, enhances cell-type discrimination and contributes to the overall performance of ModelistsGCN.

### Ablation and sensitivity analyses

We next performed systematic ablation analyses to quantify the contribution of individual components of the ModelistsGCN framework. Removal of loss function terms (Table S6) shows that all components contribute to performance, with the propagation loss having the largest effect. Evaluation of GMM initialization strategies (Table S7) demonstrates that modelist-guided initialization improves agreement with expected cell types, with unsupervised initialization increasing Silhouette score (~16%) but reducing ARI (~30%), indicating reduced biological coherence. Targeted ablation of graph construction (Table S8) shows that disrupting spatial organization or gene expression markedly reduces performance, while removing or shuffling morphology also decreases ARI, supporting a contribution of morphological features.

Complementary sensitivity analyses further demonstrate robustness to parameter variation and input noise. ModelistsGCN maintains stable performance under reduced modelist cell utilization and moderate marker corruption (Fig. S11), as well as under moderate reductions in gene coverage (Table S9). Additional biological validation of clusters lacking predefined modelist anchors using differential expression and enrichment analysis further supports robustness of inferred cell identities (Table S10). In addition, cell–cell communication analysis provides complementary validation of the inferred cell identities and supports the biological interpretability of ModelistsGCN annotations (Fig. S12). Varying the spatial neighborhood radius used for graph construction (i.e. the distance threshold defining edges between neighboring cells) over a broad range (5–15 μm) results in only modest changes in performance, despite substantial differences in graph connectivity (Tables S11–S12). Similarly, performance remains stable across a range of loss weight configurations, with most variations within approximately 10% of default performance, and larger effects observed primarily when removing key components such as the propagation loss (Figs. S13–S14). Together, these analyses support that ModelistsGCN is robust, with multiple components contributing to performance while maintaining stability under realistic perturbations.

## Discussion

Spatially resolved transcriptomics provides new opportunities to study gene expression within intact tissues; however, accurate single-cell cell-type identification remains challenging due to the limited transcript coverage relative to standard single-cell sequencing approaches [14]. Here, we present ModelistsGCN, a semi-supervised graph convolutional framework that integrates gene expression with morphological features and spatial proximity to improve cell-type inference in single-cell spatial transcriptomic datasets.

A key feature of this framework is the use of modelist cells, defined as high-confidence exemplars based on robust marker gene expression, to initialize the Gaussian mixture prior. This biologically guided initialization anchors clustering to expected cellular identities and mitigates instability that can arise in fully unsupervised settings. As a result, ModelistsGCN performs reliably under limited gene coverage, including the 300-gene ExSeq, MERFISH and Xenium datasets analysed here. By incorporating limited prior knowledge directly into the model, the framework bridges supervised cell typing and unsupervised clustering, enabling biologically guided and spatially informed assignments even when only a subset of cells can be confidently annotated. We note that, in the absence of ground-truth labels for the metastatic breast cancer datasets analysed here, evaluation based on marker coherence and agreement with modelist annotations is not fully independent of the priors used by the model; however, we mitigate this limitation by (i) validating performance on a ground-truth annotated dataset (mouse visual cortex), (ii) restricting modelist and marker information in the model to a small subset of high-confidence cells used for initialization, while evaluation assesses overall marker enrichment and cluster-level coherence, and (iii) adopting evaluation metrics that are standard in the field and consistently applied to competing methods, enabling fair and comparable evaluation.

While ModelistsGCN offers several advantages, certain considerations highlight opportunities for further extension. The framework relies on the availability of a small set of marker-informed exemplar cells to guide initialization. In contexts where prior marker knowledge is sparse or less well defined, this guidance may be less informative, suggesting that complementary strategies for identifying high-confidence exemplars could further broaden applicability. In addition, ModelistsGCN incorporates morphological features across the datasets analysed here; however, the relevance of specific morphological descriptors can vary with tissue type, disease context, and imaging protocol. Morphological features were defined in 3D to capture cell geometry across z-planes, which is increasingly relevant for image-based spatial transcriptomics platforms that support multi-plane and thick tissue imaging. Adapting or learning morphology features in a data-driven manner may further improve performance across diverse experimental settings. Furthermore, improvements in cell segmentation are expected to directly enhance single-cell cell typing performance. More accurate delineation of cell boundaries, for example by incorporating membrane information, as demonstrated here, would improve the quality of extracted morphological descriptors and reduce misassignment of RNA transcripts located between adjacent nuclei [31]. Recent advances in foundation models for single-cell analysis (e.g. scDrugMap [32]) and deep learning approaches for image-based phenotypic profiling (e.g. PhenoProfiler [33]) highlight the potential of leveraging related data modalities and pretrained models to further improve spatial cell typing.

To conclude, ModelistsGCN improves cell-type inference in spatial transcriptomics under limited gene coverage by jointly integrating gene expression, spatial proximity, and morphological information within a graph-based framework. By leveraging modelist cells to provide biologically guided initialization of a learned Gaussian mixture prior, the method produces coherent and interpretable cell-type assignments even when only a small subset of cells can be confidently annotated. Across ExSeq and MERFISH datasets, ModelistsGCN consistently yielded more compact and better separated clusters, reflected by higher silhouette scores, along with stronger marker-gene enrichment relative to established graph-based approaches. Together, these results highlight ModelistsGCN as a framework for cell-type inference in single-cell spatial transcriptomic data with limited gene coverage.

**Key Points**

- Sparse gene panels in single-cell spatial transcriptomics limit transcriptome-based cell typing accuracy.
- ModelistsGCN integrates gene expression, spatial proximity, and quantitative 3D morphology within a graph neural network framework.
- A small set of biologically defined ‘modelist’ cells guides clustering under limited transcriptomic coverage.
- The model jointly optimizes graph embedding and Gaussian mixture clustering for stable, biologically coherent assignments.
- ModelistsGCN improves clustering agreement, marker enrichment, and embedding separation across ExSeq and MERFISH datasets, and generalizes to Xenium data.

## Supplementary Material

Supplementary_Data1_bbag340

Supplementary_Data2_bbag340

Supplementary_Data3_bbag340

## Data Availability

ModelistsGCN is publicly available: https://github.com/NoaKonforti/ModelistsGCN, and archived at https://doi.org/10.5281/zenodo.18651263. A tutorial and a test dataset for running the code, together with example output, are provided in the GitHub repository and mirrored in the Zenodo archive.
