# Supplementary material for: ModelistsGCN: a multimodal graph convolutional network framework for single-cell spatial transcriptomic cell typing
Source: Brief Bioinform. 2026 Jun 22;27(3):bbag340. doi: 10.1093/bib/bbag340 (PMC13284712; doi:10.1093/bib/bbag340)
Supplement: Supplementary_Data1_bbag340 [file supplementary_data1_bbag340.pdf]

Supplementary Methods

Ground-truth dataset: curated ExSeq mouse visual cortex

To establish a reliable reference for supervised training and validation, a set of consensus-labeled cells from an ExSeq dataset of the mouse visual cortex was curated. This dataset was analyzed in two independent studies: one provided raw spatial sequencing data with gene-expression-based cell-type annotations[1], while the other provided harmonized cell-type labels obtained by integrating results from multiple computational tools[2]. Cell typing was originally performed using Seurat[3-4] in[1], and the labels reported in[2] were derived from an ensemble of six different algorithms (ATLAS[5], FR-Match[6], map.cells[7], mfishtools[8], pciSeq[9], and Tangram[10]). Cell segmentation of the ExSeq data was performed using the tool VAST[11] using the DAPI images[1].

Because the two studies [1-2] differed in segmentation strategies and clustering granularity, corresponding cells were registered based on spatial position and morphological similarity. This alignment yielded 948 overlapping cells, of which 276 showed consistent cortical layer cell-type assignments. To expand beyond this set, we leveraged the expression of cortical layer-specific marker genes (L2/3: "Cux2", "Lamp5", "Cxcl14";L4: "Rorb", "Rspo1";L5: "Fezf2", "Parm1"; L6: "Sema3e", "Foxp2", "Syt6";[2]), retaining cells in the top 25th percentile of marker expression, provided that their assignments did not conflict with other marker-based annotations. This procedure added 289 cells, yielding a final set of 565 consensus-labeled cells.

These consensus-labeled cells, spanning cortical layer-specific and other known cell types, served as high-confidence labels for training and evaluation of the GCN. After filtering out the least variable 5% of genes, 39 of the original 42 measured genes[1-2] were retained for downstream analysis. The ExSeq dataset was generated with approximately 3.3× expansion; however, all distances and measurements are reported in pre-expansion units.

Breast cancer tissues

To assess generalizability beyond the curated ground-truth set, we analyzed 15 additional tissues from[12], comprising five ExSeq metastatic breast cancer tissues and ten MERFISH metastatic breast cancer tissues. Unlike the visual cortex dataset, these datasets do not include ground-truth cell-type labels. The ExSeq and MERFISH datasets differed in scale: ExSeq metastatic breast cancer tissues contained between 386 and 2,575 cells per sample, whereas MERFISH tissues included between 3,414 and 9,480 cells. In all tissues, the same 300-gene panel was measured, providing a consistent feature space across platforms.

Cell segmentation for the MERFISH data was performed using Cellpose[13] via its Python package (https://github.com/MouseLand/cellpose), applying the 'nuclei' model to DAPI images[12]. For the ExSeq data, segmentation was performed using InSituSeg[14], also based on DAPI images. As before, the ExSeq data were generated with approximately 3.3× expansion; however, all distances and measurements are reported in pre-expansion units. These unlabeled datasets were used to benchmark unsupervised and spatially informed cell-type inference in the absence of ground-truth labels.

Morphological feature extraction

To capture morphological characteristics of cells, we extracted the following features from 3D cell segmentation data, based on methodologies established in prior literature[15–17]:

- Volume: Computed as the total voxel count enclosed by the cell shape.
- Surface area: Estimated by reconstructing the outer surface of each 3D cell segmentation and computing the total surface area using mesh-based calculations (implemented with scikit-image[18]).
- Shape compactness: Measure how efficiently a cell's volume is enclosed relative to its surface area and spatial extent $\frac{V}{A\left(L_x L_y L_z\right)^{1/3}}$.
- Elongation ratio: Defined as $1-\frac{d_{\min}}{d_{\max}}$, with values approaching 1 for elongated cells and 0 for isotropic cells.

- Sphericity: Quantifies the degree to which a cell's shape approaches that of a perfect sphere via $\frac{\pi^{1/3}(6V)^{2/3}}{A}$.

Where V is the volume, A is the surface area, L_x equals $\max(x) - \min(x)$ and similarly for L_y and L_z, and $d_{\min}$ and $d_{\max}$ are the minimum and maximum cell diameters, respectively.

Detecting neighboring cells

We identified neighboring cells based on a minimum inter-cell distance threshold of 10 μm, corresponding to a typical cell-scale neighborhood. Cell-cell distance was defined as the Euclidean distance between cell centroids. To approximate a 10 μm minimum boundary-to-boundary distance, we applied a 20 μm centroid-to-centroid threshold, assuming an average centroid-to-boundary distance of approximately 5 μm per cell, consistent with typical compact cell morphology. At this threshold, proximate cells showed higher transcriptional similarity than distant cells (Fig. 2a, and parameter sensitivity tests in Fig. S1).

Building graph representations for single cell spatial data

We constructed a graph representation of the single-cell spatial transcriptomic data by modeling each cell as a node and connecting pairs of cells that reside in close spatial proximity (see "Detecting neighboring cells"). For connected cell pairs, edge weights were defined as the inverse of the Euclidean distance between their centroids, thereby assigning greater weight to nearby cells. Node features consist of a concatenated feature vector comprising z-scored gene expression and morphological features described above. This graph formulation explicitly encodes spatial relationships between cells. During training, information can propagate through the graph via the loss functions described below, allowing node representations to reflect not only local immediate cellular neighbors but also wider cellular neighborhoods.

To test whether applying PCA to the gene expression and morphological features improves performance, we trained a GCN classifier, using the GCN architecture detailed in the Methods section "Graph encoder" below, on the ground-truth dataset using either the original features or PCA-reduced features. Performance was

comparable in both cases, indicating that PCA did not provide a measurable advantage (Fig. S1c).

We next evaluated the contribution of spatial edges and node features by retraining the same GCN classifier after shuffling either the graph connectivity or the gene expression and morphological features. In both cases, performance was significantly reduced relative to the original model (KS test, $p < 10^{-8}$), confirming that both spatial proximity and cell-intrinsic features are required for accurate classification (Fig. S1d).

Graph encoder

The GCN uses a multi-layer graph convolutional encoder implemented with PyTorch Geometric[19] to integrate gene expression, morphological, and spatial information into a shared latent representation. The encoder operates on the spatial graph defined above and performs localized message passing using the normalized graph convolution formulation of Kipf and Welling[20]. ModelistsGCN employs a single hidden GCN layer with 64 units, followed by a latent layer of 16 dimensions.

Rectified linear unit (ReLU) activations and dropout (dropout probability = 0.3) were applied after each graph convolutional layer to reduce overfitting during training. The encoder outputs a latent embedding matrix $Z \in \mathbb{R}^{N \times 16}$, where N denotes the number of cells and each row corresponds to a 16-dimensional latent representation of a single cell. Model training was performed using the Adam optimizer[21] with a learning rate of 0.01 for 20 epochs, with a fixed random seed of 5507.

Marker genes selection

Marker genes were curated using a data-driven strategy based on existing cell-type annotations from a reference scRNA-seq dataset[12], generated from consecutive tissue sections of the same metastatic breast cancer samples analyzed by ExSeq and MERFISH. Gene expression profiles were normalized and scaled across all genes. Differential expression analysis was performed using Seurat[3-4] to identify genes enriched in each annotated cell type. Genes were retained as candidate markers if they showed strong and specific enrichment, defined by an average $\log_2$ fold change > 5 and extremely significant differential expression (adjusted $p \approx 0$; in Seurat outputs this corresponds in practice to adjusted $p \leq 1 \times 10^{-200}$). To ensure marker specificity and avoid ambiguous assignments, genes detected as significant

markers in more than one cluster were conservatively filtered. As a result, only genes uniquely enriched in a single cluster were retained. These data-derived markers were complemented with a curated reference panel of canonical cell-type markers from[14], including established markers for T cells, B cells, macrophages, fibroblasts, and tumor cells. The final marker panel was defined as the union of metastatic breast cancer and canonical markers (Table S3).

We note that this procedure represents one of several possible strategies for marker gene detection; alternative approaches, such as literature-based selection, cross-dataset consensus markers, or automated feature selection methods, could be substituted depending on dataset characteristics and study goals.

Modelists cell selection

To incorporate partial biological prior knowledge, we defined modelist cells as high-confidence representatives of selected expected cell types used to guide semi-supervised learning. Prior to modelist selection, expression-based quality control was performed by retaining only cells with at least 100 sequencing reads, thereby excluding low-complexity profiles that could bias marker-based cell-type assignment. Modelist cells were defined based on the expression of curated marker genes (see supplementary Methods section ‘Marker genes selection’) indicative of specific cell types (Table S3). For each expected cell type, cells were selected that overexpressed its corresponding markers, defined as expression above the 90th percentile, while simultaneously underexpressing markers of all other expected cell types, defined as expression at or below their mean levels (Fig. S2). All cells meeting this marker-specificity criterion were designated as modelist cells for the corresponding type. These modelist cells were used to initialize the Gaussian mixture model centroids and to provide label anchors during training.

Gaussian mixture clustering and initialization

Clustering in the latent space of ModelistsGCN was performed using an embedded GMM, which assigns each cell a soft probabilistic cluster membership based on its latent representation. This formulation is conceptually related to Deep Embedded Clustering[22], in which clustering is performed jointly with representation learning. In ModelistsGCN, the GMM parameters and the GCN encoder were optimized jointly

on the spatial graph, such that latent representations and cluster assignments were updated simultaneously during training.

The GMM was implemented using the GaussianMixture class from the scikit-learn library[23], with the covariance type set to *tied*, enforcing a shared covariance matrix across all mixture components. This choice reduces the number of learnable parameters and improves numerical stability. In the breast cancer samples analyzed here, the target number of clusters (K), corresponding to the final number of inferred GMM components, was set for each sample as reported in[12]. For each tissue, the specific value of K corresponds to the number of cell types listed in Table S2, excluding cells labeled as unknown (described below).

For clusters containing modelist cells, the GMM component means (μ_k) were initialized from the latent embeddings of these cells (Fig. S3). For clusters without modelist cells, an overparameterized fallback GMM, initialized with twice the target number of components, was first fitted to all cell embeddings in an unsupervised manner. Candidate GMM components whose means lay within a Euclidean distance threshold of any modelist-initialized centroid were excluded to avoid redundancy. This distance threshold was defined as the minimum pairwise distance between modelist-initialized centroids in PCA space. From the remaining components, initial means were selected using a greedy farthest-point strategy, iteratively choosing component means that maximized their distance from both modelist-initialized centroids and previously selected centroids (Fig. S3). This hybrid initialization was performed at each training epoch, after which the GMM parameters, including mixture weights, means, and the shared covariance matrix, were refined jointly with the encoder. This approach integrates prior marker-based information with latent representations learned from the data within the GMM-based clustering framework.

Label propagation and semi-supervised training

ModelistsGCN incorporates a label propagation module to diffuse label information from modelist cells to non-modelist cells over the cell-cell graph. Modelist cells act as fixed label anchors, while labels for all cells are represented as a soft label matrix $Y \in \mathbb{R}^{n_{\text{cells}} \times n_{\text{clusters}}}$.

Label propagation is performed by iteratively updating Y based on the graph cell-cell adjacency matrix A and the corresponding degree matrix D. The initial label matrix Y^0 assigns one-hot labels (unit probability for the assigned cluster and zero otherwise) for modelist cells and uniform weights across clusters for non-modelist cells. The iterative update of Y is governed by the following rule:

$$Y^{(t+1)} = \alpha D^{-1} A Y^{(t)} + (1-\alpha) Y^0$$

where α controls the extent of neighborhood influence. Propagation was performed for a fixed number of iterations. The values $\alpha = 0.8$ and five propagation iterations were selected based on simulation analyses (Figs. S4-S5). In the simulations, label assignments largely converged within five iterations, and additional iterations did not substantially change the results. A relatively high value of α was chosen to ensure effective influence from first-order neighbors. Sensitivity analyses across alternative modelist initialization patterns and graph configurations demonstrated that $\alpha = 0.8$ yielded stable behavior compared with slightly lower or higher α values. These parameters can be adjusted by the user to modulate the strength and range of label propagation. The resulting matrix Y represents soft probabilistic label assignments for all the cells.

To select cells used for semi-supervised training, label-propagation confidence was assessed using the maximum posterior assignment probability in Y. Specifically, cells satisfying:

$$\underset{k}{max} Y_{ik} > \frac{1}{n_{clusters}} + \beta$$

were included, together with modelist cells, in the propagation loss. This threshold restricts the propagation loss to cells with sufficiently confident soft assignments. The parameter β was determined using simulations (Fig. S5) and set to 0.03 for all analyses. Lower values tended to include more second-order neighbors, whereas higher values reduced inclusion of some first-order neighbors (Fig. S5); $\beta = 0.03$ provided a balanced confidence threshold. This parameter can be adjusted to increase or decrease the extent of label propagation.

During training, supervision is applied through a propagation loss that uses these selected cells to incorporate propagated label information. This extends supervision

beyond local immediate cellular neighbors to wider cellular neighborhoods, without requiring complete cell-type annotations.

Loss function

ModelistsGCN was trained using a composite loss comprising three terms: (i) a label propagation loss for semi-supervised training, (ii) a GMM-based clustering loss that encourages compact cluster structure in the latent space, and (iii) a contrastive loss that leverages feature-space similarity and modelist labels to encourage similarity between embeddings of related cells and separation of unrelated cells. The total loss was defined as a weighted sum of the three loss terms:

$$\mathcal{L} = \alpha \mathcal{L}_{prop} + \beta \mathcal{L}_{gmm} + \gamma \mathcal{L}_{con}$$

Adjusting the loss term weights allows different aspects of the training objective to be emphasized and can be tuned by users of ModelistsGCN. Here, the propagation and contrastive losses were equally weighted ($\alpha = \gamma = 1$). As detailed below, the GMM loss promotes geometric compactness of clusters in the latent space; however, increasing the weight of this term places greater emphasis on cluster compactness in the embedding. To balance cluster compactness with data-driven structure, the GMM pull loss was assigned a lower weight ($\beta = 0.6$). Similar performance was observed for values of β in the range 0.5-0.7.

- Propagation loss:
  This loss term allows label information from a small set of confidently labeled cells to be propagated to nearby cells in the spatial graph, providing supervision beyond the initial anchors.
  To apply supervision beyond the modelist anchors, we use the propagated soft label matrix Y as training targets. For each cell i, the propagated label vector $\tilde{y}_i$ corresponds to the i-th row of Y. Given per-cell cluster logits $s_i \in \mathbb{R}^K$ derived from the GMM head, and a supervision mask m_i indicating modelist cells and propagated cells with high posterior assignment probability in Y (as defined in the "Label propagation and semi-supervised training" section above), the propagation loss is defined as the cross-entropy between

propagated soft labels and the model predictions:

$$\mathcal{L}_{\text{prop}} = -\frac{1}{|M|} \sum_{i \in M} \sum_{k=1}^{K} \tilde{y}_{ik} \log(\text{softmax}(s_i)_k), \quad M = \{i : m_i = 1\}.$$

- GMM pull loss:

  This loss term encourages cells assigned to the same mixture component to form compact groups in the latent space by pulling their embeddings toward the corresponding component centers.

  Let $\mu_k \in \mathbb{R}^d$ denote the current means of the Gaussian mixture model (GMM) components, obtained from the fitted GMM in latent space.

  We first ℓ_2-normalize both cell embeddings and GMM means:

  $$\hat{z}_i = \frac{z_i}{\|z_i\|_2},$$

  $$\hat{\mu}_k = \frac{\mu_k}{\|\mu_k\|_2}$$

  Each cell embedding is assigned to the GMM component whose normalized mean has the highest dot product with the normalized embedding, equivalent to maximizing cosine similarity:

  $$c_i = \arg \max_{k \in \{1,\ldots,K\}} \hat{z}_i^\top \hat{\mu}_k$$

  The GMM pull loss encourages each normalized embedding $\hat{z}_i$ to align with its assigned normalized component mean $\hat{\mu}_{c_i}$ by penalizing one minus the cosine similarity:

  $$\mathcal{L}_{\text{gmm}} = \frac{1}{N} \sum_{i=1}^{N} \left(1 - \cos(\hat{z}_i, \hat{\mu}_{c_i})\right).$$

- Contrastive Loss:

  To encourage similarity between embeddings of related cells and separation of unrelated cells in the latent space, we incorporate a contrastive objective. Positive and negative cell pairs are defined based on cosine similarity in PCA space of the input features (gene expression and morphology): pairs above the 85th percentile are treated as positives, and pairs below the 15th percentile as negatives. In addition, pairs of modelist cells sharing the same label are included as positive pairs, while pairs of modelist cells with different labels are treated as negative pairs.

The contrastive loss is formulated as a binary classification objective over pairwise similarities in embedding space:

$$\mathcal{L}_{\text{con}} = \frac{1}{|\mathcal{V}|} \sum_{(i,j)\in\mathcal{V}} \text{BCE}\left(\frac{\hat{z}_i^\top \hat{z}_j}{T}, t_{ij}\right).$$

where BCE denotes the binary cross-entropy loss, $\hat{z}_i = \frac{z_i}{\|z_i\|_2}$ denotes the normalized embedding of cell i, $t_{ij} \in \{0,1\}$ indicates whether the pair (i,j) is a positive or negative pair, $T$is a temperature hyperparameter, and $\mathcal{V}$ denotes the set of valid positive and negative pairs used to compute the contrastive loss.

Cell-type annotations

In the absence of ground-truth cell-type annotations, biological identities were assigned to clusters using cluster-wise differential expression analysis. For each cluster, cells belonging to that cluster were compared against all remaining cells using DESeq2[24]. Raw gene-count matrices (cells × genes) were used as input, and differential expression was computed separately for each cluster. Genes that passed multiple-testing correction ($q < 1 \times 10^{-3}$) and showed a positive $\log_2$ fold change were ranked by q value. For each cluster, the three most significantly upregulated genes were selected to guide inference of putative cell identities. These genes were cross-referenced against information from the Human Protein Atlas ‘Cell type RNA expression’ and ‘Cancer & cell lines’ resources to determine whether they are cell-type-enriched or cancer-enhanced, respectively. Clusters showing coherent enrichment of cell-type-enriched genes from a single lineage were considered biologically consistent, whereas clusters with mixed signatures or signatures not annotated as cell-type-enriched or cancer-enhanced in the Human Protein Atlas were classified as unknown. This classification enabled assessment of whether each cluster’s transcriptional profile was consistent with a recognizable cell type. These assignments are referred to as “predicted cluster assignments” in the Methods sections below.

Evaluation of clustering without ground-truth labels

In the absence of ground-truth cell-type annotations, clustering performance was evaluated using a combination of embedding-level separation metrics and marker gene-based biological consistency measures:

1. Silhouette score:
   The silhouette score was computed for each cell typing method using its corresponding representation space. For ModelistsGCN, embeddings were obtained from the GCN encoder. For SpaGCN and GraphST, embeddings were extracted from their respective implementations[25-26]. Squidpy does not learn a latent embedding but performs clustering directly on precomputed features; therefore, the combined feature space used for clustering (denoted as X_combined in the Squidpy implementation[27]) was used for silhouette score computation. This metric quantifies clustering separation by comparing within-cluster cohesion and between-cluster separation within each method's representation space, with higher values indicating better-defined clusters.
2. Marker gene-based scoring:
   To quantitatively assess the biological coherence of inferred clusters, we evaluated enrichment of reference cell-type marker genes within each predicted cluster assignment. Unlike the "Cell-type annotations" Methods section above, which uses full differential expression profiles and Human Protein Atlas information to assign putative identities, this analysis was restricted to a predefined reference panel of curated marker genes (Table S3) and was used solely for scoring purposes. For each cluster, differential expression was assessed relative to all other cells, and significantly upregulated genes were identified after multiple-testing correction ($q < 1 \times 10^{-3}$). Enrichment of reference marker genes was then quantified using marker recall and marker precision metrics.
   - Marker Recall (MR; completeness of expected markers recovered): For each cluster, marker recall was defined as the fraction of reference marker genes corresponding to the assigned cell type that were identified as significantly upregulated in that cluster. Higher MR values indicate more complete recovery of cell-type-specific marker genes.
   - Marker Precision (MP; specificity of markers within a cluster): For each cluster, marker precision was defined as the fraction of significantly

upregulated marker genes corresponding to the assigned cell type, relative to all significantly upregulated marker genes associated with any cell type. Higher MP values indicate greater specificity of the cluster to the assigned cell type.

   - F1 Score: Marker recall and marker precision were combined using their harmonic mean:

$$F1 = \frac{2 \cdot MR \cdot MP}{MR + MP}$$

3. ARI on modelist cells:
   ARI was computed on modelist cells by comparing predicted cluster assignments to the cell-type labels assigned during modelist selection.

Together, these metrics capture complementary aspects of clustering performance, including separation in the representation space (silhouette score), marker gene enrichment within clusters (MR and MP), and agreement with cell-type labels on a high-confidence subset of cells (ARI on modelist cells).

Input and output of ModelistsGCN

ModelistsGCN operates on single-cell spatial transcriptomics data and requires the following inputs:

1) Gene expression matrix (CSV format): a cell-by-gene count matrix where:
   - Rows correspond to individual cells (unique cellID)
   - Columns correspond to measured genes
   - Values represent raw gene expression counts

   This matrix defines the molecular feature space of the cells.

2) Marker gene for expected cell types (CSV format): a table specifying marker genes for expected cell types. The table should include:
   - At least two expected cell types (clusters) are required. Providing three or more expected cell types improves performance and is therefore recommended.

- At least one marker gene per cell type is required. Providing four or more marker genes per cell type improves performance and is therefore recommended.

These markers are used to identify high-confidence modelist cells, which anchor Gaussian mixture component initialization and guide semi-supervised clustering.

3) Number of clusters (K): users specify the total number of clusters to infer, which determines the final number of Gaussian mixture components learned in the latent space. When K cannot be estimated a priori, ModelistsGCN can be run with multiple K values to assess robustness.

4) Spatial and morphological inputs (two alternative modes)
ModelistsGCN supports two alternative input configurations for incorporating spatial and morphological information:
Choose either (a) or (b):
a) Segmentation-based mode
Cell segmentation mask (NumPy .npy file): a 3D labeled image in which each voxel is assigned a cell identifier, which should match the aforementioned cellID. Label 0 denotes background. This mask is used to:
    - Define individual cell boundaries
    - Derive cell morphology
    - Compute distances between cells

From this segmentation, the pipeline automatically extracts morphological descriptors (e.g., volume, surface area, elongation, sphericity) and computes cell centroids.

b) Feature-based mode (without segmentation data)
If a segmentation mask is not provided, the following inputs are BOTH required:
1) Cell centroid coordinates:
A table of spatial coordinates used to define relationships between cells, formatted as: (cellID, centroids_z, centroids_y, centroids_x)

2) Morphological and additional optional features:
A feature table aligned by cellID, containing morphological properties and, optionally, additional descriptors such as imaging-derived statistics (e.g., mean or integrated fluorescence intensity) or functional measurements (e.g., neuronal activity).

ModelistsGCN outputs the trained model comprising the learned GCN and the fitted GMM in the latent space, together with a Pandas DataFrame containing predicted cluster assignments for all cells. The DataFrame is indexed by cellID, consistent with the original gene expression matrix, and includes a single column, *pred*, with integer cluster labels ranging from 0 to K−1, where K denotes the user-specified number of clusters. Cell-type annotation can then be performed as described in the “Cell-type annotations” Methods section above.

References

1. Alon S, Goodwin DR, Sinha A, et al. Expansion sequencing: Spatially precise in situ transcriptomics in intact biological systems. Science 2021; 371:eaax2656
2. Zhang Y, Miller JA, Park J, et al. Reference-based cell type matching of in situ image-based spatial transcriptomics data on primary visual cortex of mouse brain. Sci. Rep. 2023; 13:9567
3. Satija R, Farrell JA, Gennert D, et al. Spatial reconstruction of single-cell gene expression data. Nat. Biotechnol. 2015; 33:495–502
4. Hao Y, Stuart T, Kowalski MH, et al. Dictionary learning for integrative, multimodal and scalable single-cell analysis. Nat. Biotechnol. 2024; 42:293–304
5. Vaishnav ED. Evolution, Evolvability, Expression and Engineering (Massachusetts Institute of Technology, PhD thesis, 2022)
6. Zhang Y, Aevermann BD, Bakken TE, et al. FR-Match: robust matching of cell type clusters from single cell RNA sequencing data using the Friedman-Rafsky non-parametric test. Brief. Bioinform. 2021; 22:bbaa339
7. Tasic B, Yao Z, Graybuck LT, et al. Shared and distinct transcriptomic cell types across neocortical areas. Nature 2018; 563:72–78
8. Nicovich PR, Taormina MJ, Baker CA, et al. Multimodal cell type correspondence by intersectional mFISH in intact tissues. bioRxiv 2019; https://doi.org/10.1101/525451
9. Qian X, Harris KD, Hauling T, et al. Probabilistic cell typing enables fine mapping of closely related cell types in situ. Nat. Methods 2020; 17:101–106
10. Biancalani T, Scalia G, Buffoni L, et al. Deep learning and alignment of spatially resolved single-cell transcriptomes with Tangram. Nat. Methods 2021; 18:1352–1362
11. Berger DR, Seung HS, Lichtman JW. VAST (Volume Annotation and Segmentation Tool): Efficient Manual and Semi-Automatic Labeling of Large 3D Image Stacks. Front. Neural Circuits 2018; 12:88
12. Klughammer J, Abravanel DL, Segerstolpe Å, et al. A multi-modal single-cell and spatial expression map of metastatic breast cancer biopsies across clinicopathological features. Nat. Med. 2024; 30:3236–3249
13. Stringer C, Wang T, Michaelos M, et al. Cellpose: a generalist algorithm for cellular segmentation. Nat. Methods 2021; 18:100–106
14. Danino-Levi M, Goldberg T, Keter M, et al. Computational analysis of super-resolved in situ sequencing data reveals genes modified by immune-tumor contact events. RNA 2024; 30:749–759
15. Bhaskar D, Lee D, Knútsdóttir H, et al. A methodology for morphological feature extraction and unsupervised cell classification. bioRxiv 2019; https://doi.org/10.1101/623793
16. Cayuela López A, Gómez-Pedrero JA, Blanco AMO, et al. Cell-TypeAnalyzer: A flexible Fiji/ImageJ plugin to classify cells according to user-defined criteria. Biol. Imaging 2022; 2:e5
17. Guan G, Chen Y, Wang H, et al. Characterizing cellular physiological states with three-dimensional shape descriptors for cell membranes. Membranes 2024; 14:137
18. van der Walt S, Schönberger JL, Nunez-Iglesias J, et al. scikit-image: image processing in Python. PeerJ 2014; 2:e453
19. Fey M, Lenssen JE. Fast graph representation learning with PyTorch Geometric. arXiv 2019; https://doi.org/10.48550/arXiv.1903.02428
20. Kipf TN, Welling M. Semi-supervised classification with graph convolutional networks. arXiv 2016; https://doi.org/10.48550/arXiv.1609.02907
21. Kingma DP, Ba J. Adam: A method for stochastic optimization. arXiv 2014; https://doi.org/10.48550/arXiv.1412.6980
22. Xie J, Girshick R, Farhadi A. Unsupervised deep embedding for clustering analysis. arXiv 2015; https://doi.org/10.48550/arXiv.1511.06335

23. Pedregosa F, Varoquaux G, Gramfort A, et al. Scikit-learn: Machine Learning in Python. J. Mach. Learn. Res. 2011; 12:2825–2830
24. Love MI, Huber W, Anders S. Moderated estimation of fold change and dispersion for RNA-seq data with DESeq2. Genome Biol. 2014; 15:550
25. Hu J, Li X, Coleman K, et al. SpaGCN: Integrating gene expression, spatial location and histology to identify spatial domains and spatially variable genes by graph convolutional network. Nat. Methods 2021; 18:1342–1351
26. Long Y, Ang KS, Li M, et al. Spatially informed clustering, integration, and deconvolution of spatial transcriptomics with GraphST. Nat. Commun. 2023; 14:1155
27. Palla G, Spitzer H, Klein M, et al. Squidpy: a scalable framework for spatial omics analysis. Nat. Methods 2022; 19:171–178
