# Supplementary material for: ModelistsGCN: a multimodal graph convolutional network framework for single-cell spatial transcriptomic cell typing
Source: Brief Bioinform. 2026 Jun 22;27(3):bbag340. doi: 10.1093/bib/bbag340 (PMC13284712; doi:10.1093/bib/bbag340)
Supplement: Supplementary_Data2_bbag340 [file supplementary_data2_bbag340.pdf]

Supplementary Figures

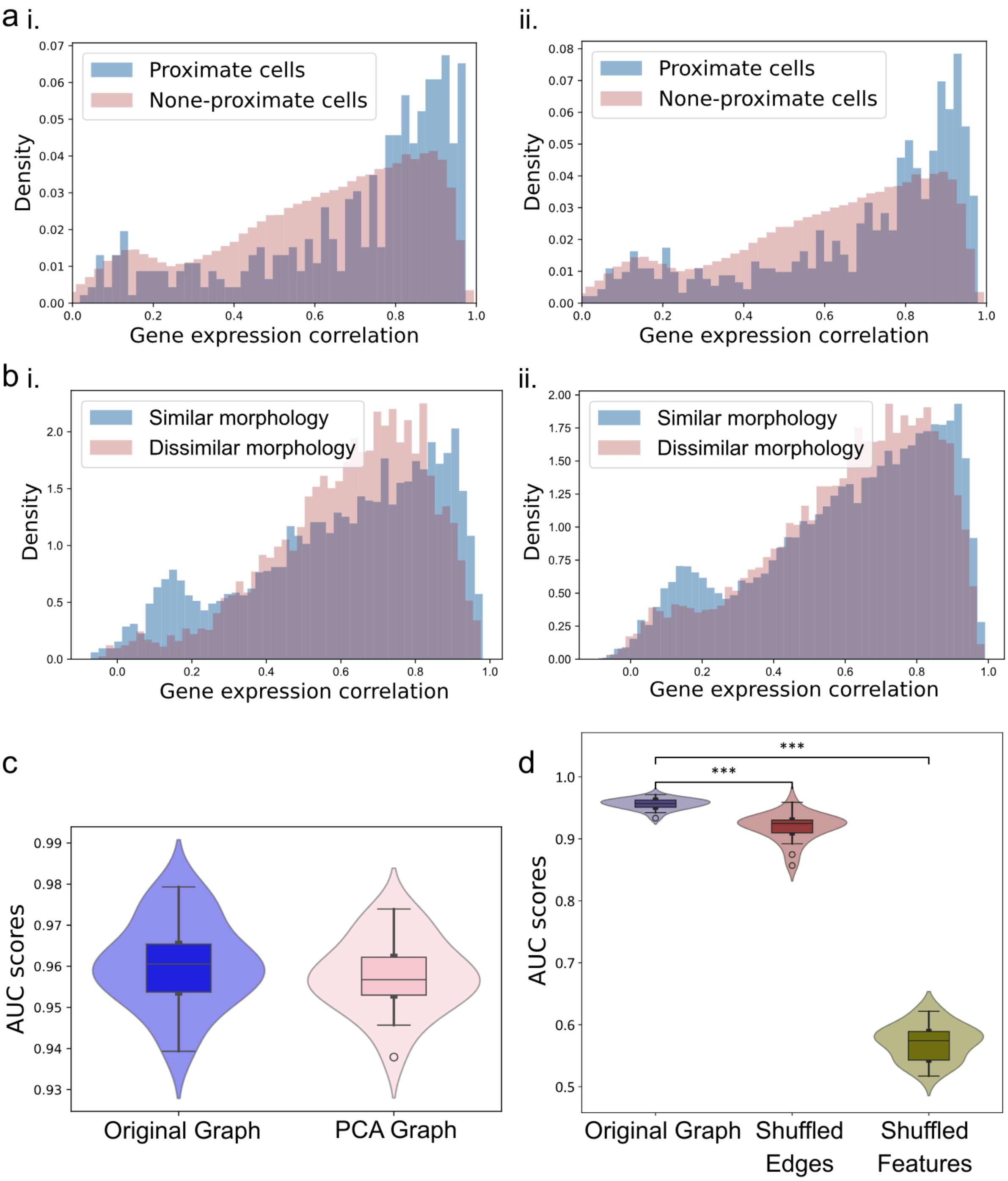

Fig. S1. Sensitivity and control analyses for spatial proximity, morphology, and feature representation. All tests in this figure were performed on the ExSeq mouse visual cortex data. **(a-b)** Parameter sensitivity tests for spatial proximity and morphology. **(a)** Distribution of pairwise gene expression correlations for proximate and non-proximate cell pairs under different neighborhood distance thresholds: (i) 5 μm (KS test, p=1.19 × 10^{-20}) and (ii) 15 μm (KS test, p=2.32 × 10^{-34}). In both cases, proximate cells show a shift toward higher correlation values, indicating increased transcriptional similarity among spatial neighbors. **(b)** Same analysis as in (a), but for pairs of cells classified as morphologically similar or dissimilar. Morphological similarity was quantified using the first principal component of the morphological feature space, with the top and bottom x% of pairwise distances defined as similar and dissimilar, respectively. x = 2.5 in (i) (KS test, p=9.6 × 10^{-13}) and x = 10 in (ii) (KS test, p=6.7 × 10^{-16}). Pairs of cells with similar morphological features exhibited higher gene expression correlations than pairs with dissimilar morphology. **(c)** Comparison of GCN classifier performance using original features versus PCA-reduced features derived from the first seven principal components. Performance distributions did not differ significantly (KS test, p = 0.83), indicating no impact of PCA-based dimensionality reduction (Methods). **(d)** Control analysis using the same classifier as in (c) after shuffling (i) graph edges (encoding cell-cell distances) and (ii) node features (expression and morphological features). In both cases, performance distributions differed significantly from the original model (edge shuffle: KS test, p = 1.13 × 10^{-8}; node feature shuffle: KS test, p = 1.45 × 10^{-11}), indicating that both spatial proximity and cell features contribute substantially to performance.

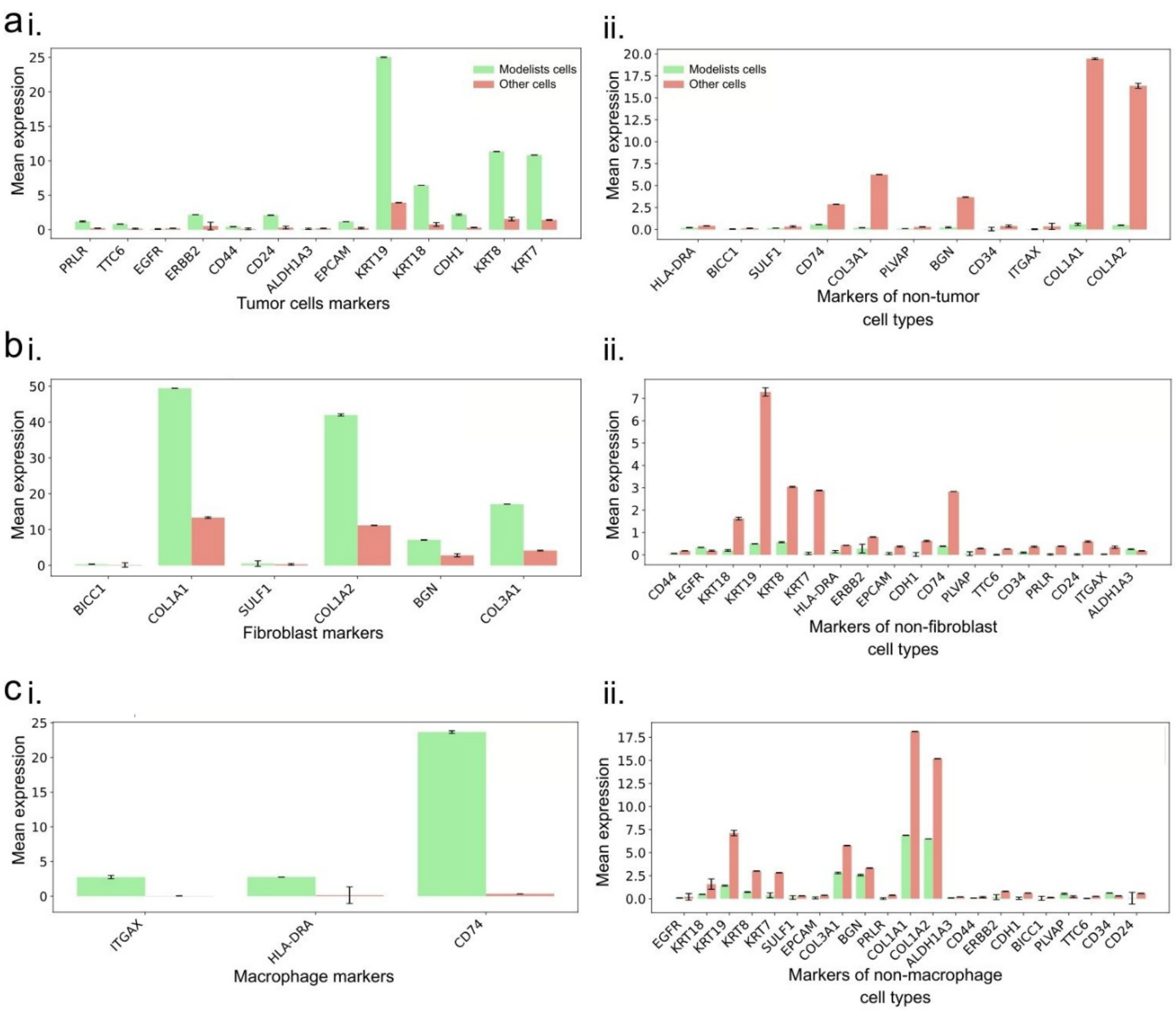
a
i.
ii.
Modelists cells
Other cells
Mean expression
PRLR
TTC6
EGFR
ERBB2
CD44
CD24
ALDH1A3
EPCAM
KRT19
KRT18
CDH1
KRT8
KRT7
Tumor cells markers
HLA-DRA
BICC1
SULF1
CD74
COL3A1
PLVAP
BGN
CD34
ITGAX
COL1A1
COL1A2
Markers of non-tumor cell types
b
Fibroblast markers
Markers of non-fibroblast cell types
c
Macrophage markers
Markers of non-macrophage cell types

Fig. S2. Validation of modelist cell selection. Marker-gene expression profiles were used to define high-confidence modelist cells for each predefined cell type. Shown are representative examples of marker-gene expression in modelist cells for three cell types in tissue “MERFISH-878”: tumor **(a)**, fibroblast **(b)**, and macrophage **(c)**. Bar plots display mean expression ± s.e.m. of cell-type-specific markers in modelist cells (green) compared with all other cells (red). For each cell type, canonical positive markers are enriched in the corresponding modelist population (i), whereas markers associated with other cell types show low expression (ii), demonstrating the specificity of the modelist selection.

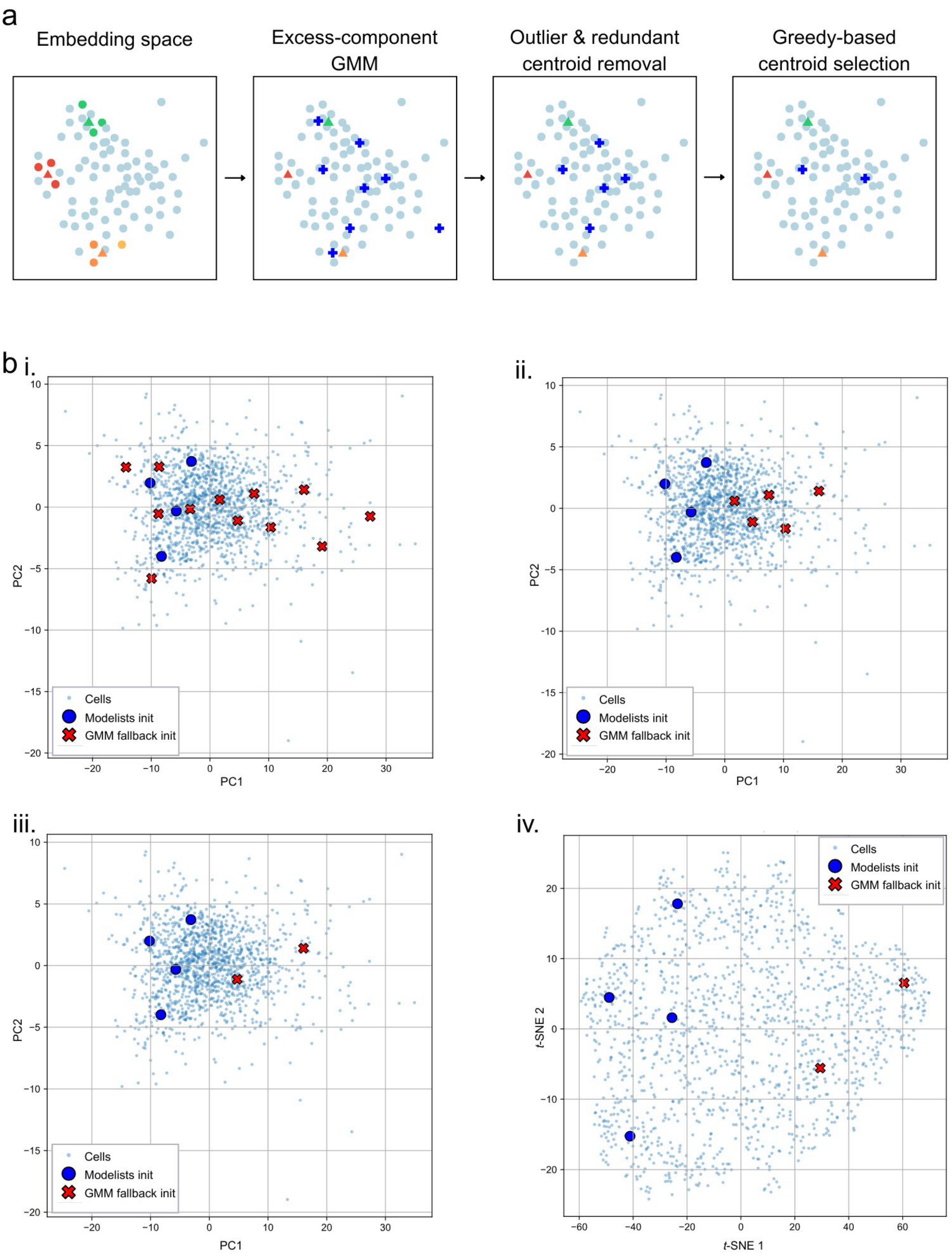

a
Embedding space
Excess-component GMM
Outlier & redundant centroid removal
Greedy-based centroid selection
b
i.
ii.
iii.
iv.
PC1
PC2
t-SNE 1
t-SNE 2
Cells
Modelists init
GMM fallback init

Fig. S3. GMM initialization. (a) Workflow for GMM mean initialization. Initial centroids are set using embeddings of modelist cells as biological anchors. To initialize the remaining centroids, a temporary GMM with more components than required is fitted to all cell embeddings to generate candidate centers. Components close to existing centroids or identified as outliers are removed, and a well-separated subset of the remaining candidates across the latent space is selected using greedy farthest-point sampling. All cells are shown as light blue circles. Modelist cells are shown in different colors according to cell type, triangles denote the mean embedding of modelist cells, and dark blue plus symbols indicate the GMM centroids. **(b)** Visualization of the GMM initialization process in PCA and *t*-SNE space for tissue "ExSeq-982". Cells are shown in light blue and modelist-initialized centroids in blue. Candidate centroids derived from a temporary GMM with more components than required (fallback GMM) are shown in red. (i) Temporary GMM with excess components fitted in PCA space. (ii) Removal of redundant and outlier components; only the remaining candidate centroids are shown. (iii) Final selection of well-separated centroids across the latent space using greedy farthest-point sampling. (iv) Same as (iii), shown in *t*-SNE space, demonstrating that the selected centroids cover the cell distribution in both PCA and *t*-SNE representations.

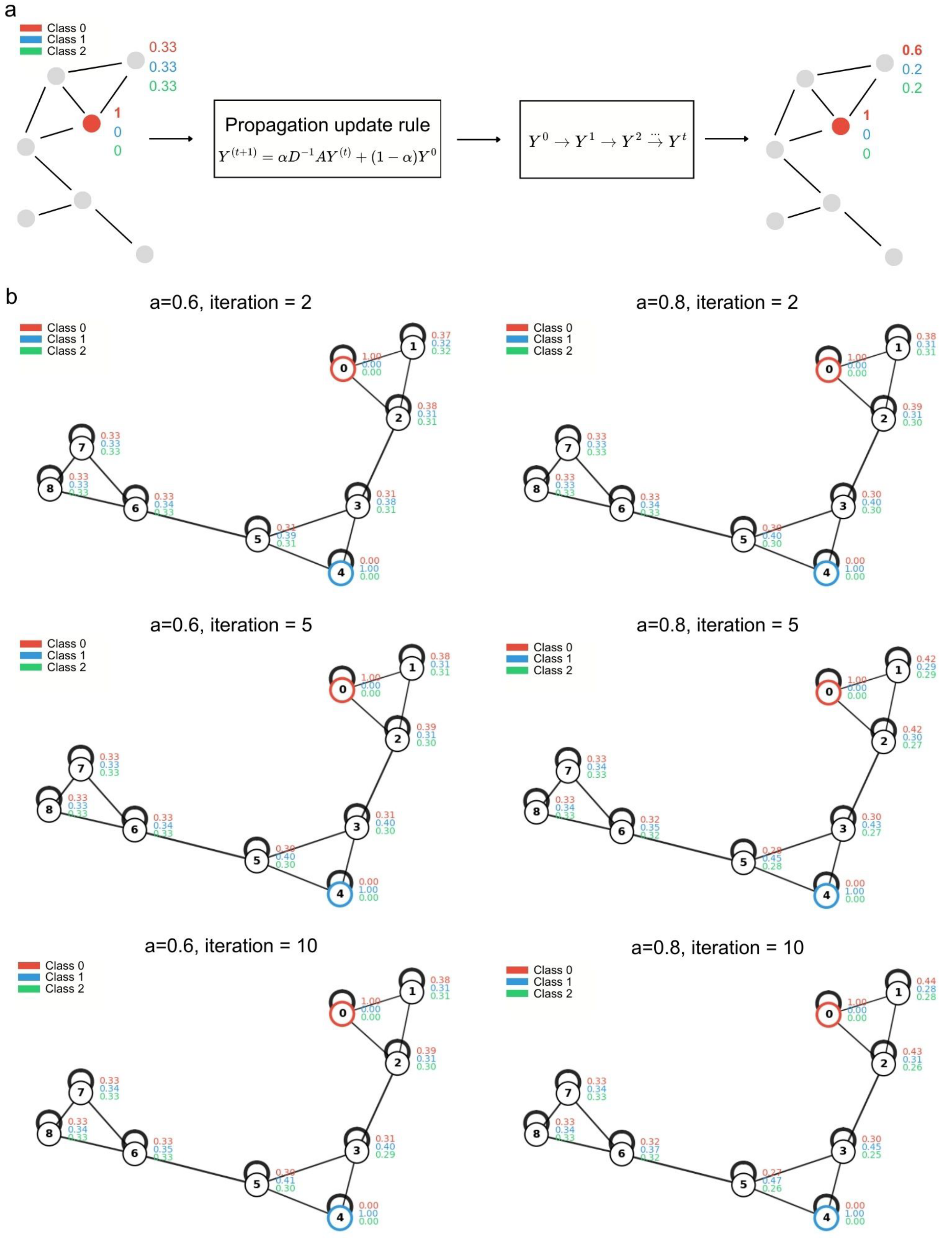
a
Class 0
Class 1
Class 2
Propagation update rule
$Y^{(t+1)} = \alpha D^{-1} A Y^{(t)} + (1-\alpha) Y^0$
$Y^0 \to Y^1 \to Y^2 \overset{\cdots}{\to} Y^t$
b
a=0.6, iteration = 2
a=0.8, iteration = 2
a=0.6, iteration = 5
a=0.8, iteration = 5
a=0.6, iteration = 10
a=0.8, iteration = 10

Fig. S4. Label propagation framework and parameter selection using simulations. (a) Schematic illustration of the label propagation process. An initial labeled cell (modelist cell, red) is embedded in a cell-cell graph together with unlabeled neighbors (gray). Soft label probabilities are iteratively updated according to the propagation update rule, resulting in gradual diffusion of label information from the labeled cell to neighboring cells over successive iterations ($Y^0 \rightarrow Y^1 \rightarrow \dots \rightarrow Y^t$). **(b)** Simulation-based evaluation of the effects of the smoothing parameter α and the number of propagation iterations on label diffusion. Black circles mark self-loops obtained in matrix A. Shown are examples for $\alpha = 0.6$ and $\alpha = 0.8$ over 2, 5, and 10 iterations. Based on stability of the resulting soft label assignments and controlled neighborhood influence (see also Fig. S5) $\alpha = 0.8$ and five iterations were selected for all analyses.

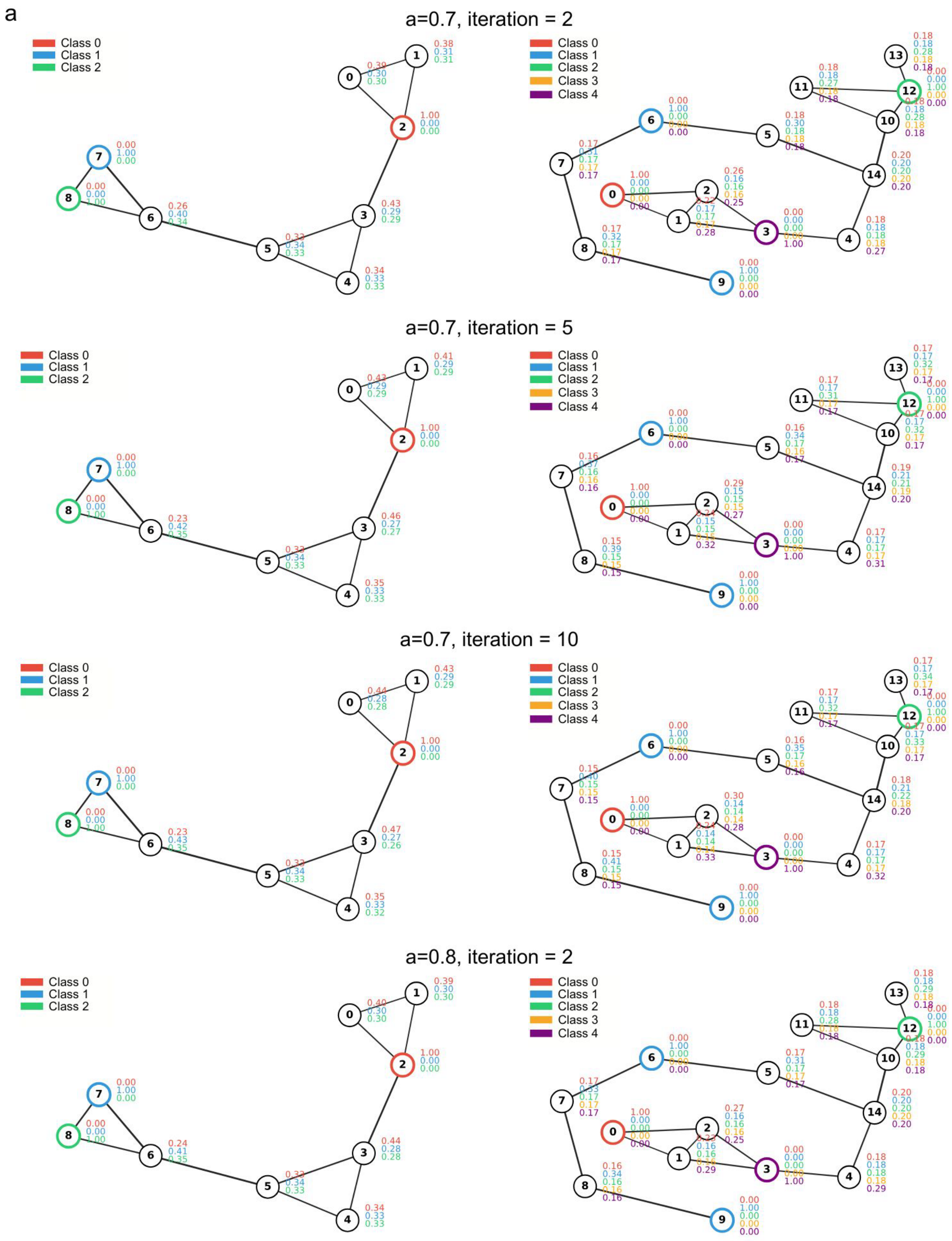
a
a=0.7, iteration = 2
a=0.7, iteration = 5
a=0.7, iteration = 10
a=0.8, iteration = 2
Class 0
Class 1
Class 2
Class 3
Class 4

a=0.8, iteration = 5

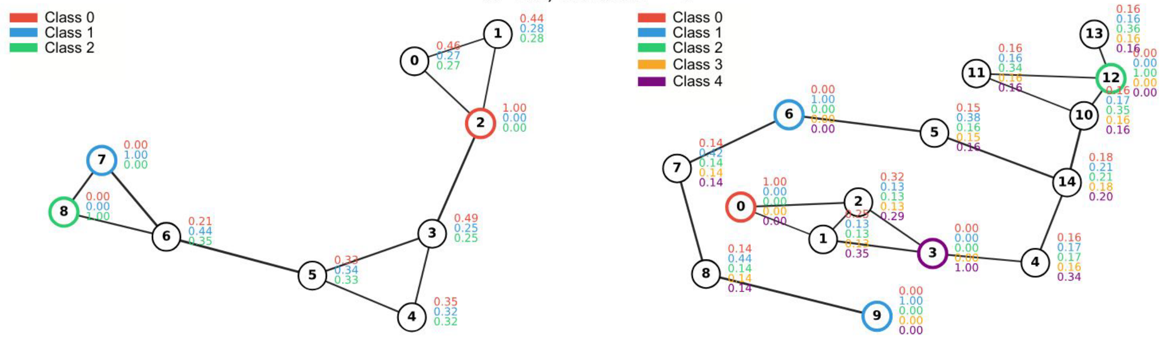

a=0.8, iteration = 10

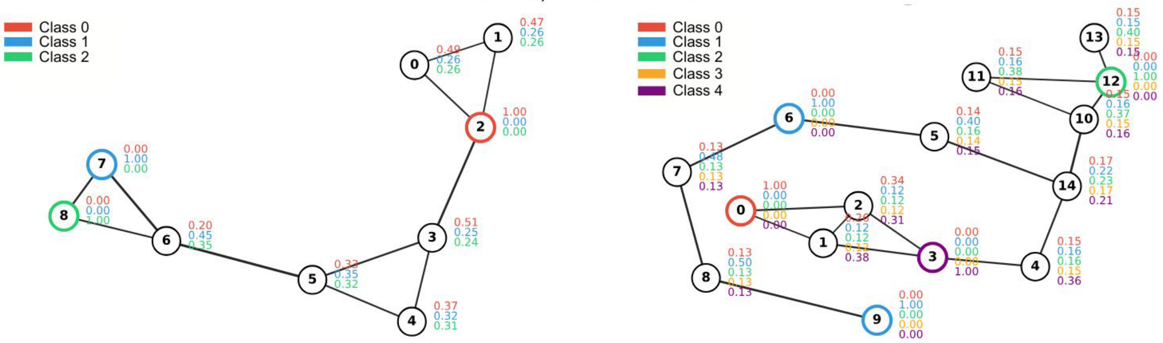

a=0.9, iteration = 2

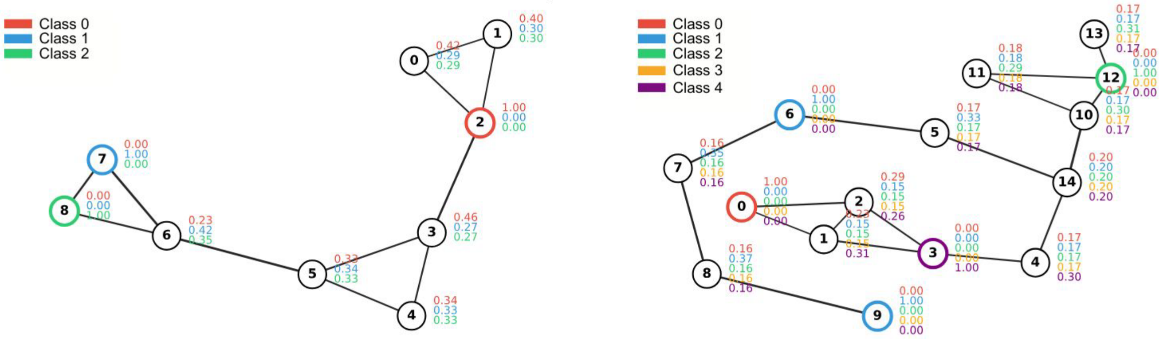

a=0.9, iteration = 5

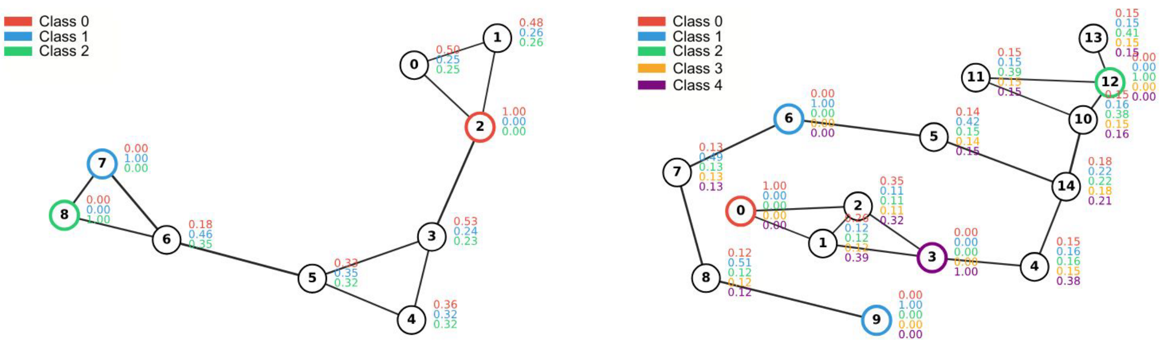

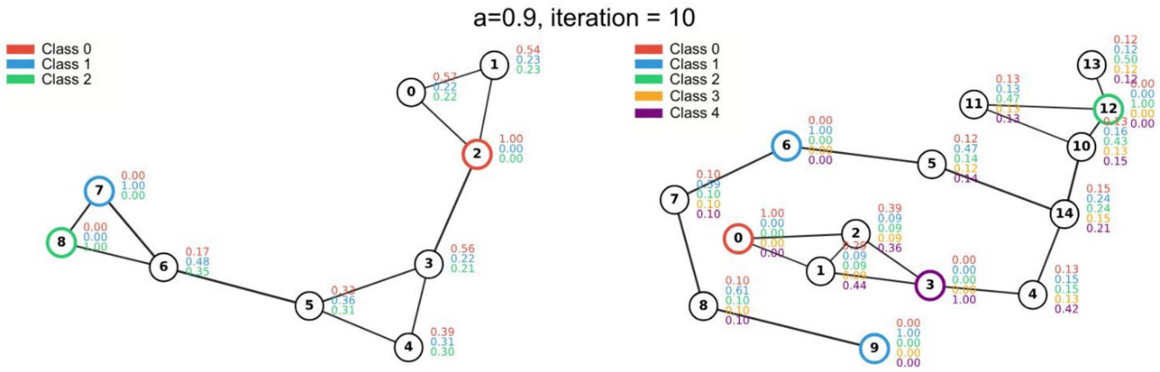
a=0.9, iteration = 10
Class 0
Class 1
Class 2
Class 0
Class 1
Class 2
Class 3
Class 4

b

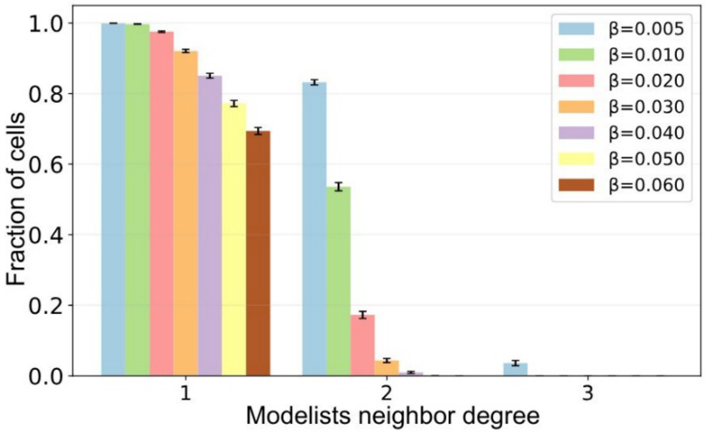
Fraction of cells
Modelists neighbor degree
β=0.005
β=0.010
β=0.020
β=0.030
β=0.040
β=0.050
β=0.060

Fig. S5. Additional simulation analyses of modelist initialization and confidence threshold selection. (a) Additional simulation examples of label propagation under alternative modelist initialization patterns and graph configurations, illustrating local diffusion of soft label probabilities from anchor cells. **(b)** Fraction of cells satisfying the confidence criterion $\underset{k}{max} Y_{ik} > \frac{1}{n_{clusters}} + \beta$ as a function of graph distance from modelist cells (neighbor degree 1–3) and β. Bars indicate mean ± standard error across simulations. The fraction of cells passing the criterion decreases with increasing distance from modelist cells and with larger β, indicating that the β-offset restricts supervision to the local neighborhood of modelist cells. To emphasize first-order neighbors, β = 0.03 was selected for all analyses. This parameter can be adjusted to increase or decrease the extent of label propagation.

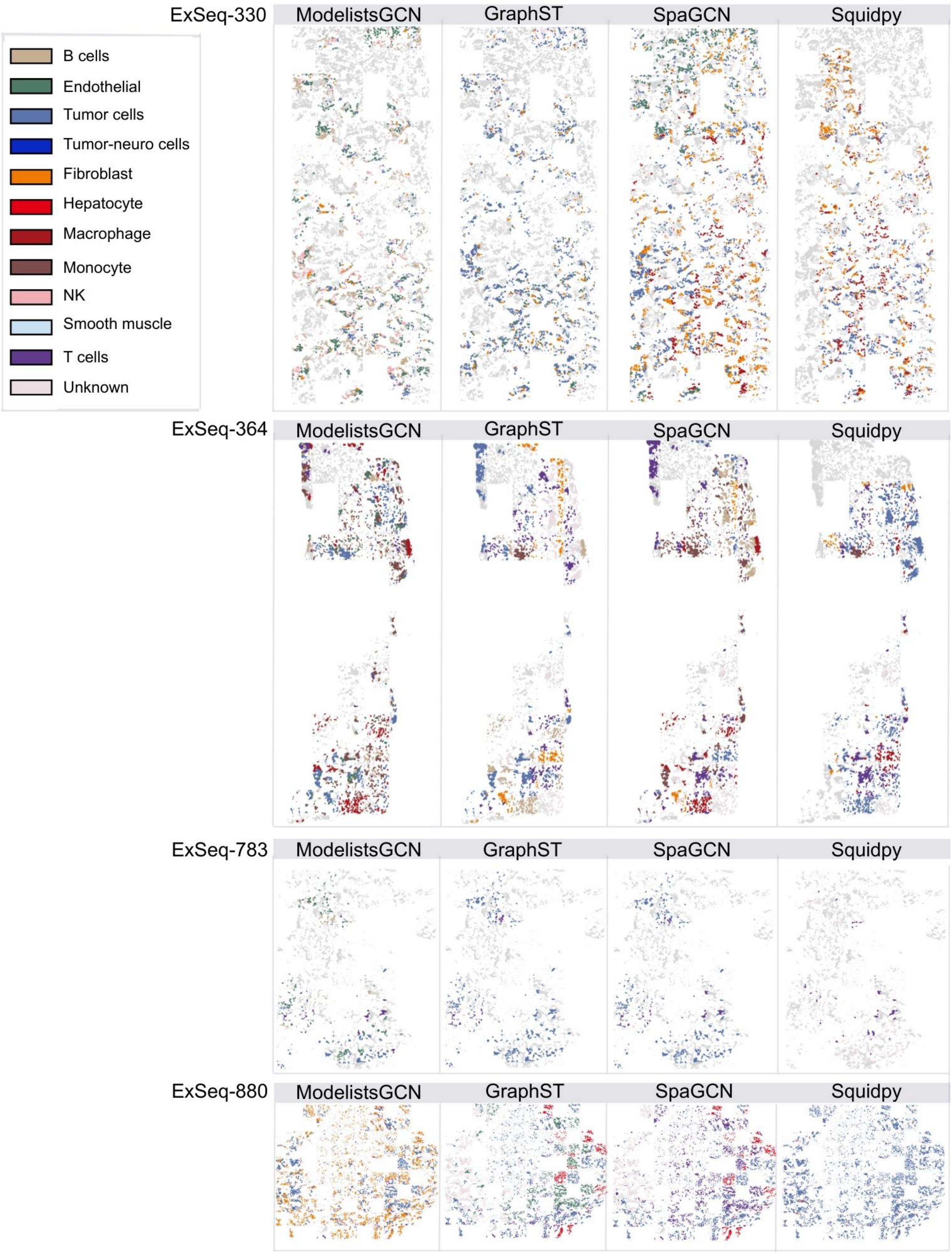
ExSeq-330
ModelistsGCN
GraphST
SpaGCN
Squidpy
B cells
Endothelial
Tumor cells
Tumor-neuro cells
Fibroblast
Hepatocyte
Macrophage
Monocyte
NK
Smooth muscle
T cells
Unknown
ExSeq-364
ModelistsGCN
GraphST
SpaGCN
Squidpy
ExSeq-783
ModelistsGCN
GraphST
SpaGCN
Squidpy
ExSeq-880
ModelistsGCN
GraphST
SpaGCN
Squidpy

Fig. S6. Spatial cell-type maps for four ExSeq tissues. Predicted cell-type assignments for ModelistsGCN, GraphST, SpaGCN, and Squidpy across four ExSeq tissues. Each dot represents a single cell, colored by its predicted cell type (legend at left); gray denotes cells assigned as unknown.

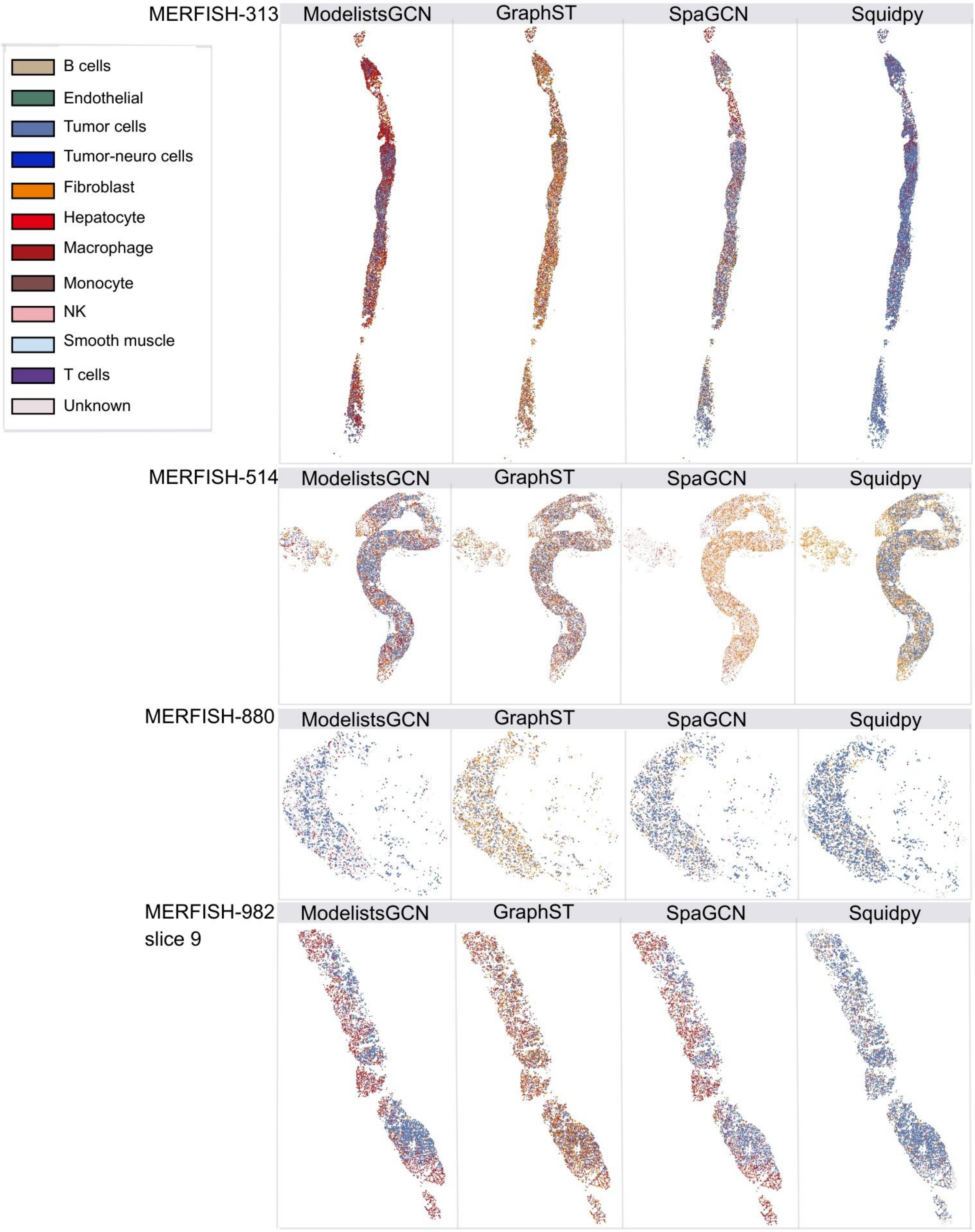

MERFISH-313
ModelistsGCN
GraphST
SpaGCN
Squidpy
B cells
Endothelial
Tumor cells
Tumor-neuro cells
Fibroblast
Hepatocyte
Macrophage
Monocyte
NK
Smooth muscle
T cells
Unknown
MERFISH-514
ModelistsGCN
GraphST
SpaGCN
Squidpy
MERFISH-880
ModelistsGCN
GraphST
SpaGCN
Squidpy
MERFISH-982
slice 9
ModelistsGCN
GraphST
SpaGCN
Squidpy

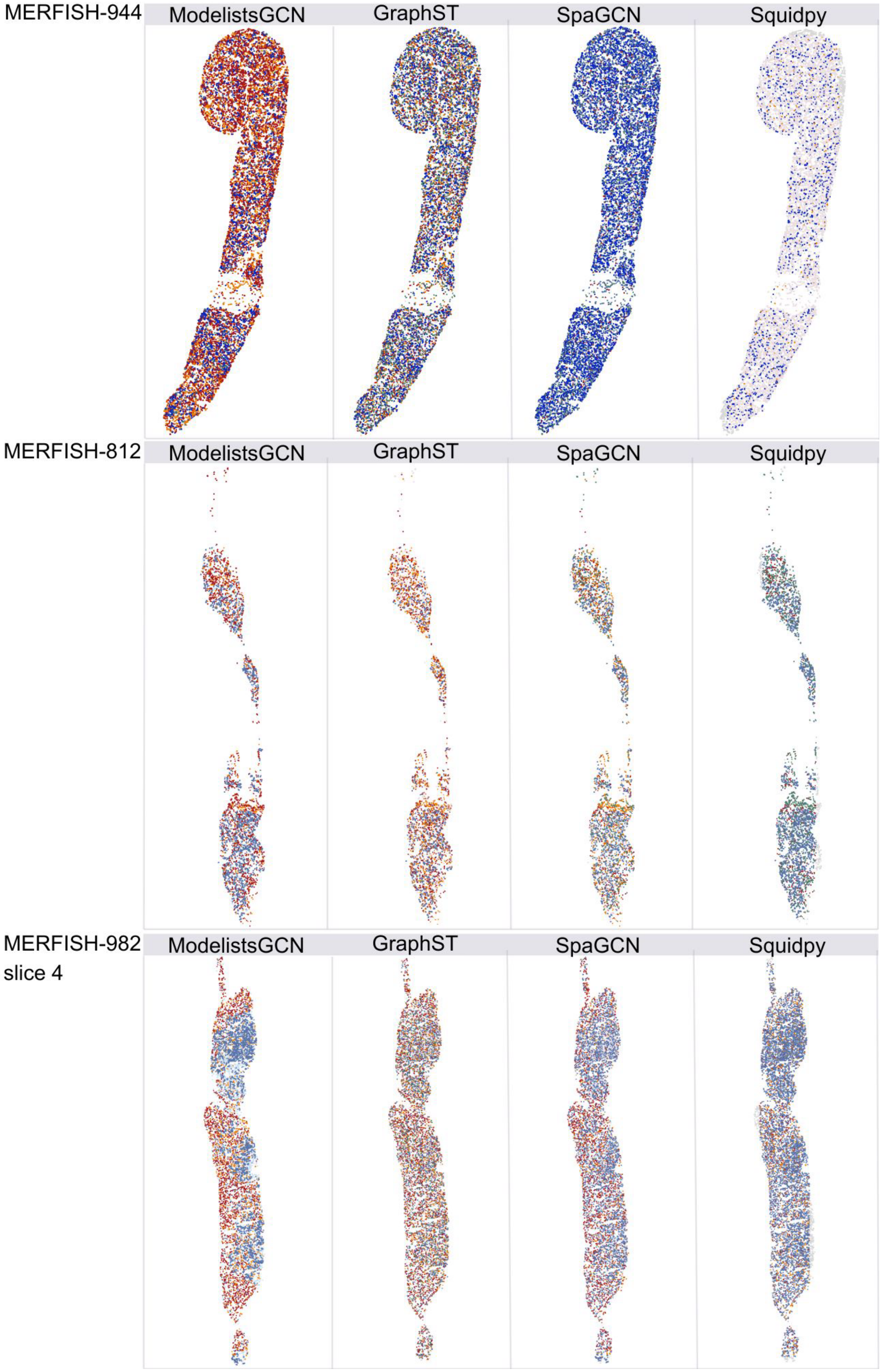
MERFISH-944
ModelistsGCN
GraphST
SpaGCN
Squidpy
MERFISH-812
ModelistsGCN
GraphST
SpaGCN
Squidpy
MERFISH-982
slice 4
ModelistsGCN
GraphST
SpaGCN
Squidpy

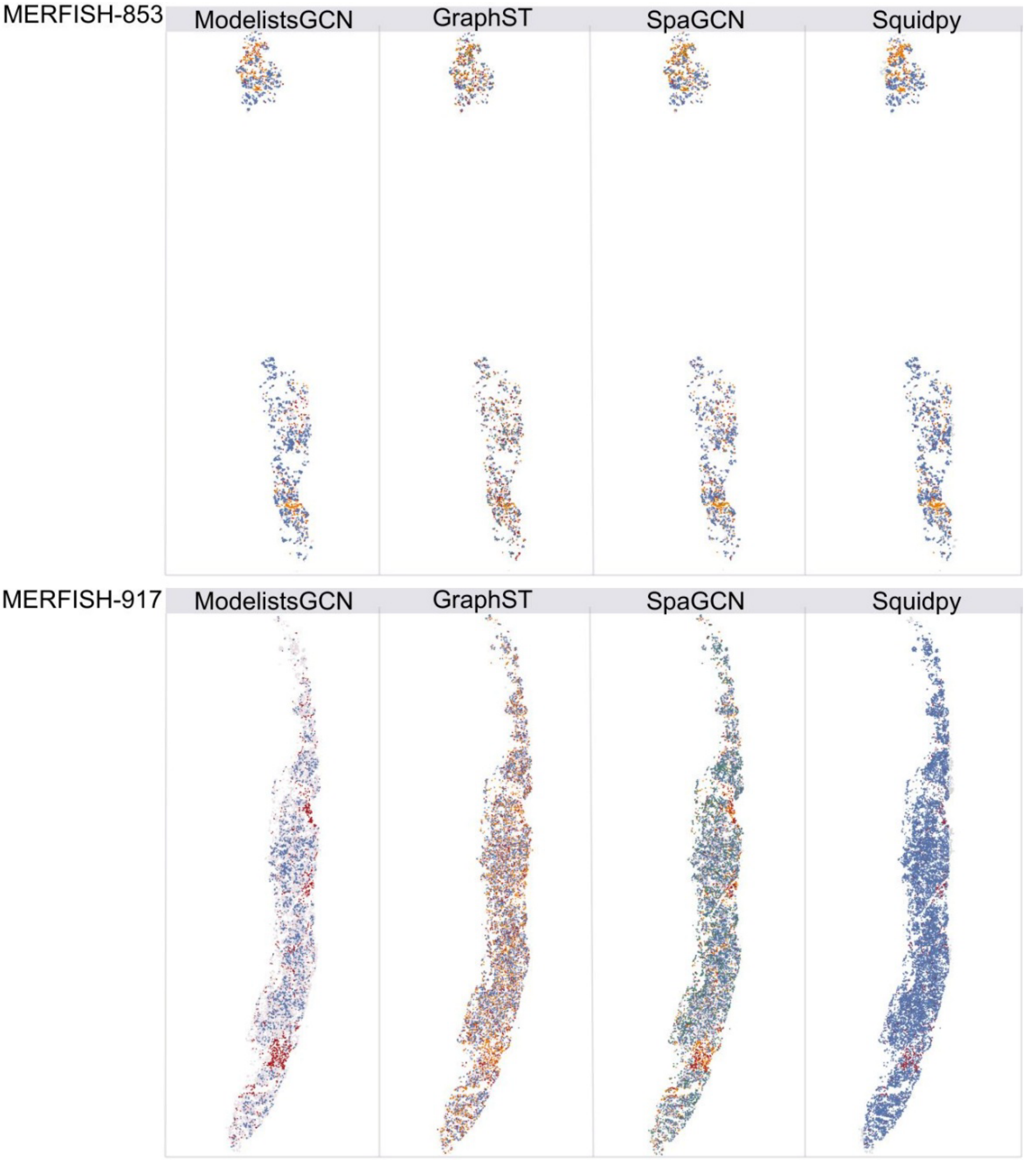

MERFISH-853
ModelistsGCN
GraphST
SpaGCN
Squidpy
MERFISH-917
ModelistsGCN
GraphST
SpaGCN
Squidpy

Fig. S7. Spatial cell-type maps for nine MERFISH tissues. Predicted cell-type assignments for ModelistsGCN, GraphST, SpaGCN, and Squidpy across nine MERFISH tissues. Each dot represents a single cell, colored by its predicted cell type (legend at left); gray denotes cells assigned as unknown.

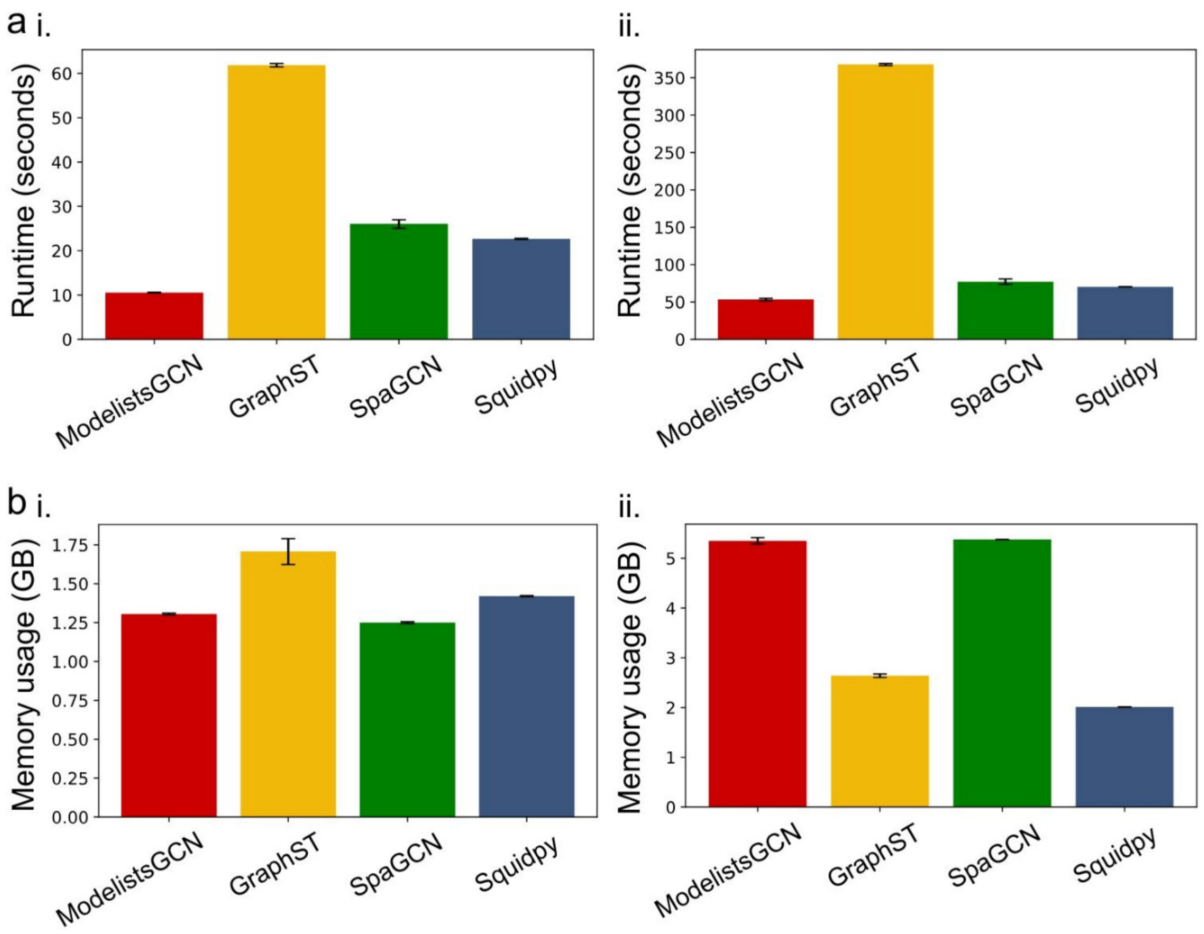
a i.
ii.
Runtime (seconds)
ModelistsGCN
GraphST
SpaGCN
Squidpy
b i.
ii.
Memory usage (GB)

Fig. S8. Computational performance comparison across spatial clustering methods. Runtime and memory usage of ModelistsGCN, SpaGCN, GraphST, and Squidpy evaluated on two MERFISH tissues. **(a)** Runtime (seconds). **(b)** Memory usage (GB). Panels (i) correspond to tissue 878 (3,414 cells), and panels (ii) correspond to tissue 944 (9,880 cells). Bars represent mean values across five independent runs under default method parameters, and error bars indicate standard error (STE). Experiments were conducted on a Linux workstation equipped with a 12th Gen Intel Core i7-12700 CPU and 32 GB RAM. These results correspond to Table S4.

a

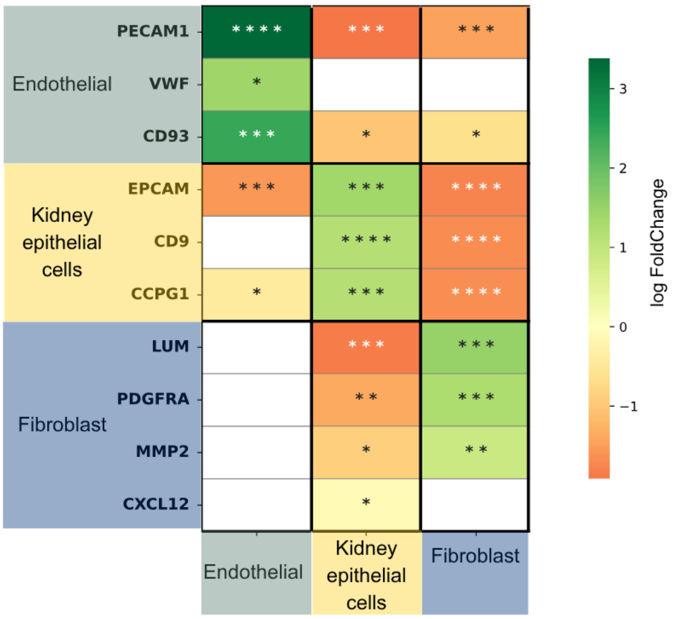

b

	Silhouette	MR	MP
ModelistsGCN	**0.301 ± 0.04**	**0.727 ± 0.02**	**0.906 ± 0.03**
SpaGCN	-0.111 ± 0.002	0.603 ± 0.02	0.875 ± 0.02

Fig. S9. ModelistsGCN performance in an additional setting using Xenium normal kidney tissue. Normal human kidney tissue profiled using Xenium (280-gene panel). Membrane stain-based segmentation was used for both ModelistsGCN and SpaGCN. **(a)** Heatmap of differential expression analysis for cell-type-specific marker genes. Green indicates upregulated genes and red indicates downregulated genes. Significance is denoted as follows: * $q < 10^{-3}$; ** $q < 10^{-12}$; *** $q < 10^{-30}$; **** $q < 10^{-100}$. **(b)** Marker-based evaluation comparing spatial cell typing methods. ModelistsGCN achieves higher Silhouette score, marker recall (MR), and marker precision (MP) compared to SpaGCN, demonstrating improved cell typing performance. This result is obtained in a setting that differs from the main datasets analyzed in this manuscript, which consist primarily of breast cancer tissues profiled using MERFISH and ExSeq, in both technology (Xenium) and tissue type (normal kidney rather than breast tumor). Values are reported as mean ± standard error across five independent runs with different random seeds. Xenium data with membrane stain-based segmentation was obtained from[1], sample identifier ‘GSM9238741_2023_Xenium’.

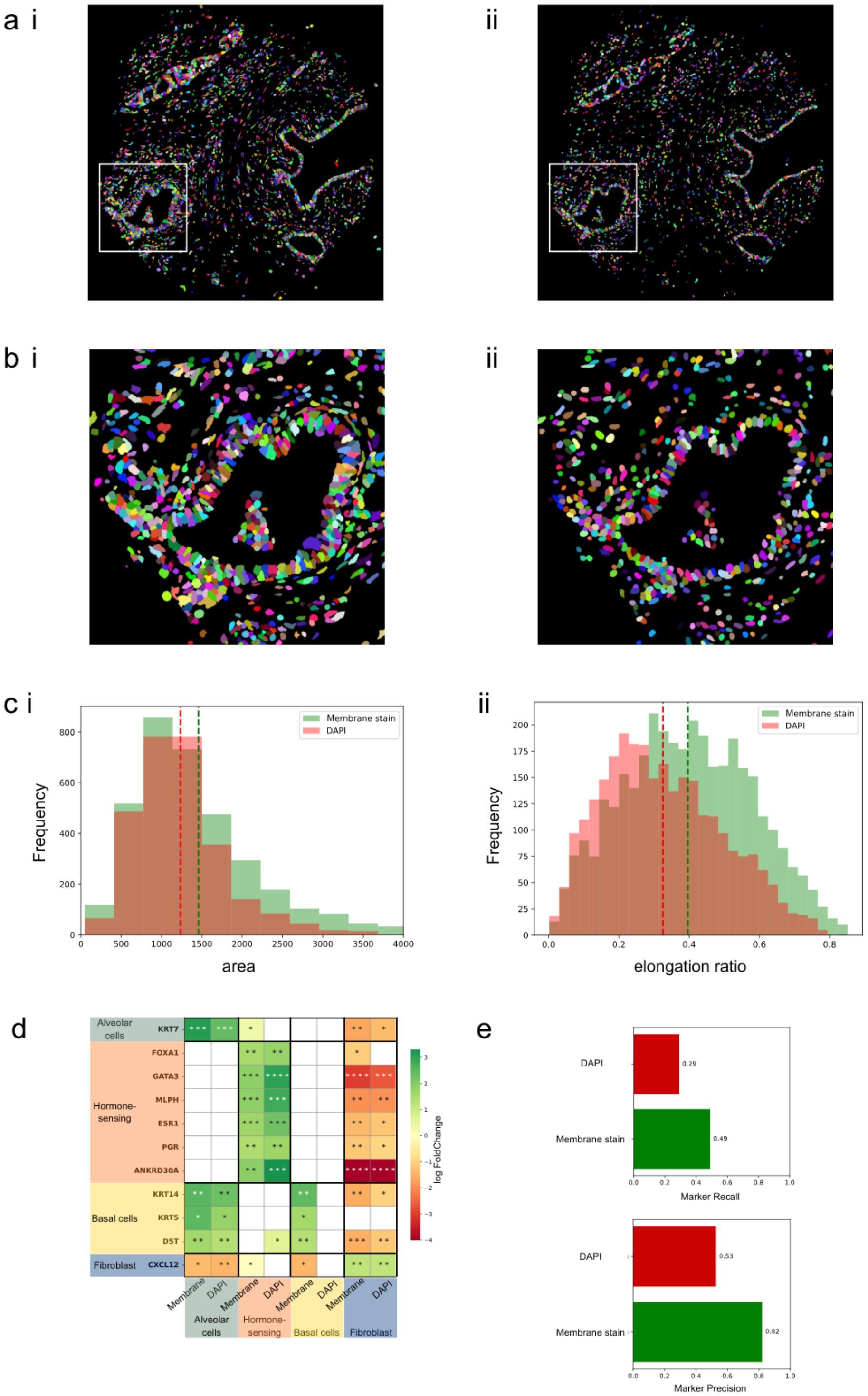
a i
ii
b i
ii
c i
Frequency
area
Membrane stain
DAPI
ii
elongation ratio
d
Alveolar cells
Hormone-sensing
Basal cells
Fibroblast
KRT7
FOXA1
GATA3
MLPH
ESR1
PGR
ANKRD30A
KRT14
KRT5
DST
CXCL12
Membrane
DAPI
log FoldChange
e
DAPI
Membrane stain
0.29
0.49
Marker Recall
0.53
0.82
Marker Precision

Fig. S10. Comparison of cell segmentation strategies and their impact on downstream analysis in a human breast cancer Xenium dataset (280-gene panel). (a) Whole-tissue views of segmented cells. (i) Segmentation based on membrane staining, where individual cell boundaries are directly resolved; (ii) segmentation based on DAPI staining using Cellpose. Each color represents a segmented cell. **(b)** Zoom-in views of the boxed regions in (a), highlighting differences in segmentation quality between membrane-based (i) and DAPI-based (ii) approaches. Membrane staining yields more continuous and complete cell boundaries, whereas DAPI-based segmentation produces smaller and more fragmented cells. **(c)** Distributions of morphological features derived from the two segmentation approaches. (i) Cell surface area and (ii) elongation ratio are both higher for membrane-based segmentation (green) compared to DAPI-based segmentation (red), consistent with more complete cell boundary delineation. **(d)** Heatmap of differential expression analysis for cell-type-specific marker genes in a single tissue, shown for both membrane- and DAPI-based segmentations. Green indicates upregulated genes and red indicates downregulated genes. Significance is denoted as follows: * $q < 10^{-3}$; ** $q < 10^{-12}$; *** $q < 10^{-30}$; **** $q < 10^{-100}$. **(e)** Marker-based evaluation comparing segmentation strategies. Membrane-based segmentation yields higher marker recall and precision compared to DAPI-based segmentation, indicating improved biological coherence of inferred cell types when cell boundaries are more accurately defined.

Xenium data with membrane stain-based segmentation and DAPI images was obtained from[1], sample identifiers: ‘GSM9238741_2023_Xenium’ and ‘GSM9238737_2024_Xenium’. Segmentation of DAPI images was performed using Cellpose[2] via its Python package (https://github.com/MouseLand/cellpose), applying the ‘nuclei’ model.

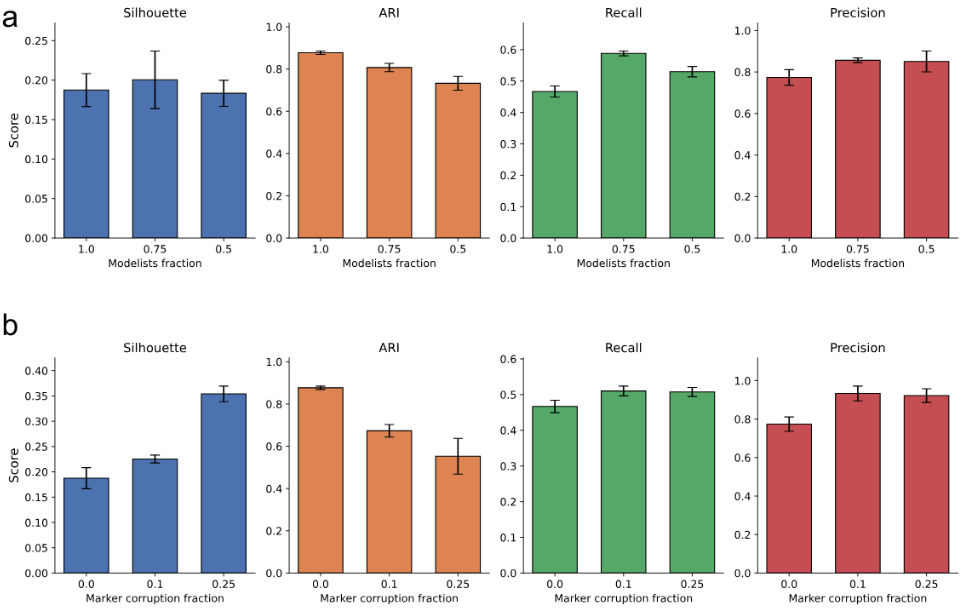

Fig. S11. Sensitivity analysis to modelist cell utilization and marker corruption using MERFISH 878 metastatic breast cancer tissue. Performance is reported as mean ± standard error (STE) across five independent runs with different random seeds. For each seed, modelist cells or marker genes were randomly subsampled or corrupted, respectively. The tissue was selected at random from the set of ten MERFISH samples analyzed in this study. **(a)** Performance as a function of the fraction of identified modelist cells used during training (1.0, 0.75, 0.5). Silhouette score, marker recall, and marker precision remain largely stable across all fractions, while ARI shows a gradual decrease (from ~0.88 to ~0.73). This indicates that ModelistsGCN is robust to partial utilization of modelist cells, with a small impact when 75% of modelist cells are used and a more pronounced, yet still moderate, reduction when only half are used. **(b)** Performance as a function of marker corruption fraction, defined as the proportion of non-marker genes introduced into the marker gene lists (0.0, 0.1, 0.25). Increasing corruption leads to a progressive decrease in ARI (from ~0.88 to ~0.55), while marker recall and precision remain relatively stable and Silhouette score is stable or increases. These results indicate that ModelistsGCN tolerates low to modest noise in marker definitions (e.g., 10% corruption), while higher noise levels reduce agreement with expected cell types.

tissue MERFISH 878

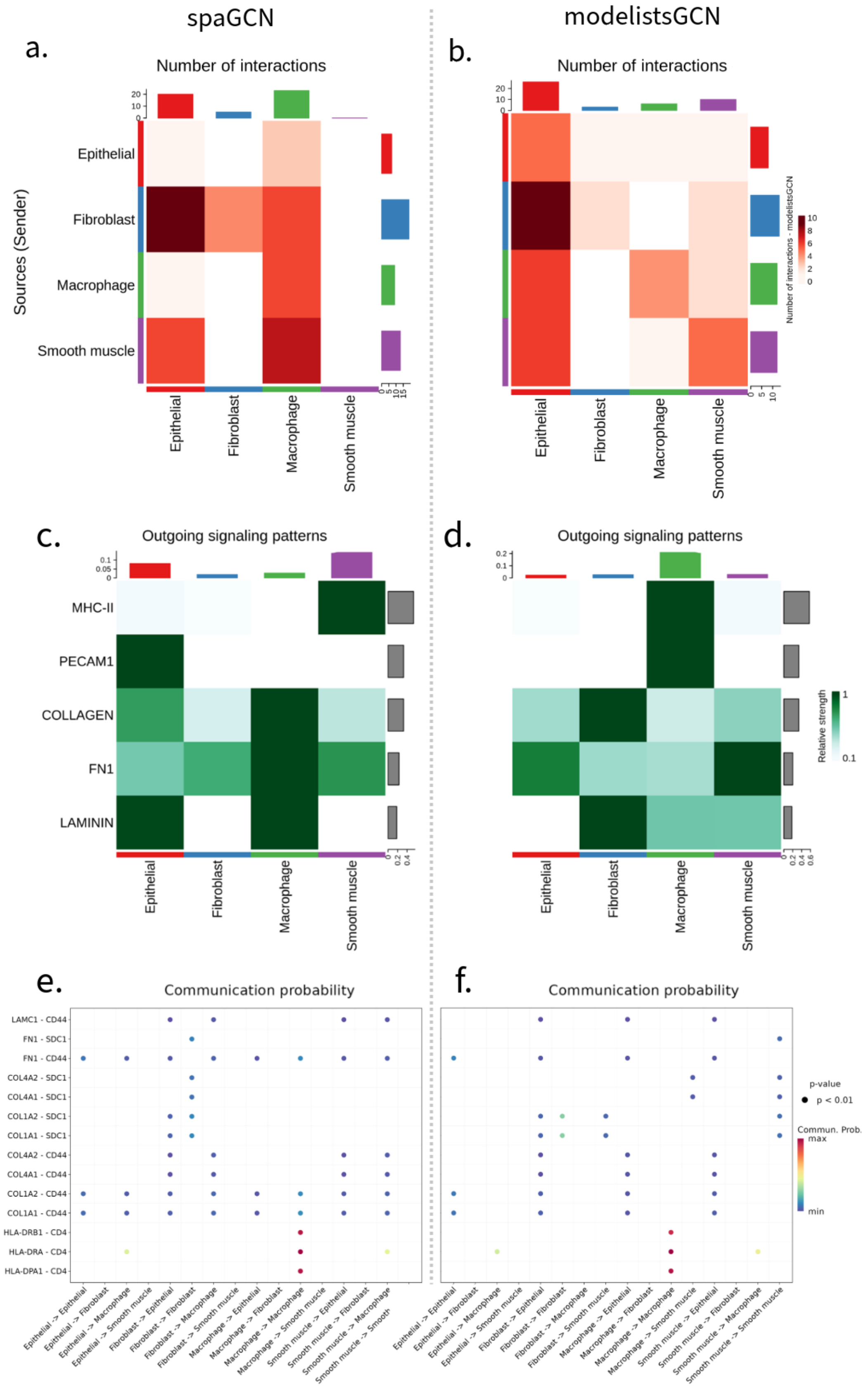

tissue MERFISH 313

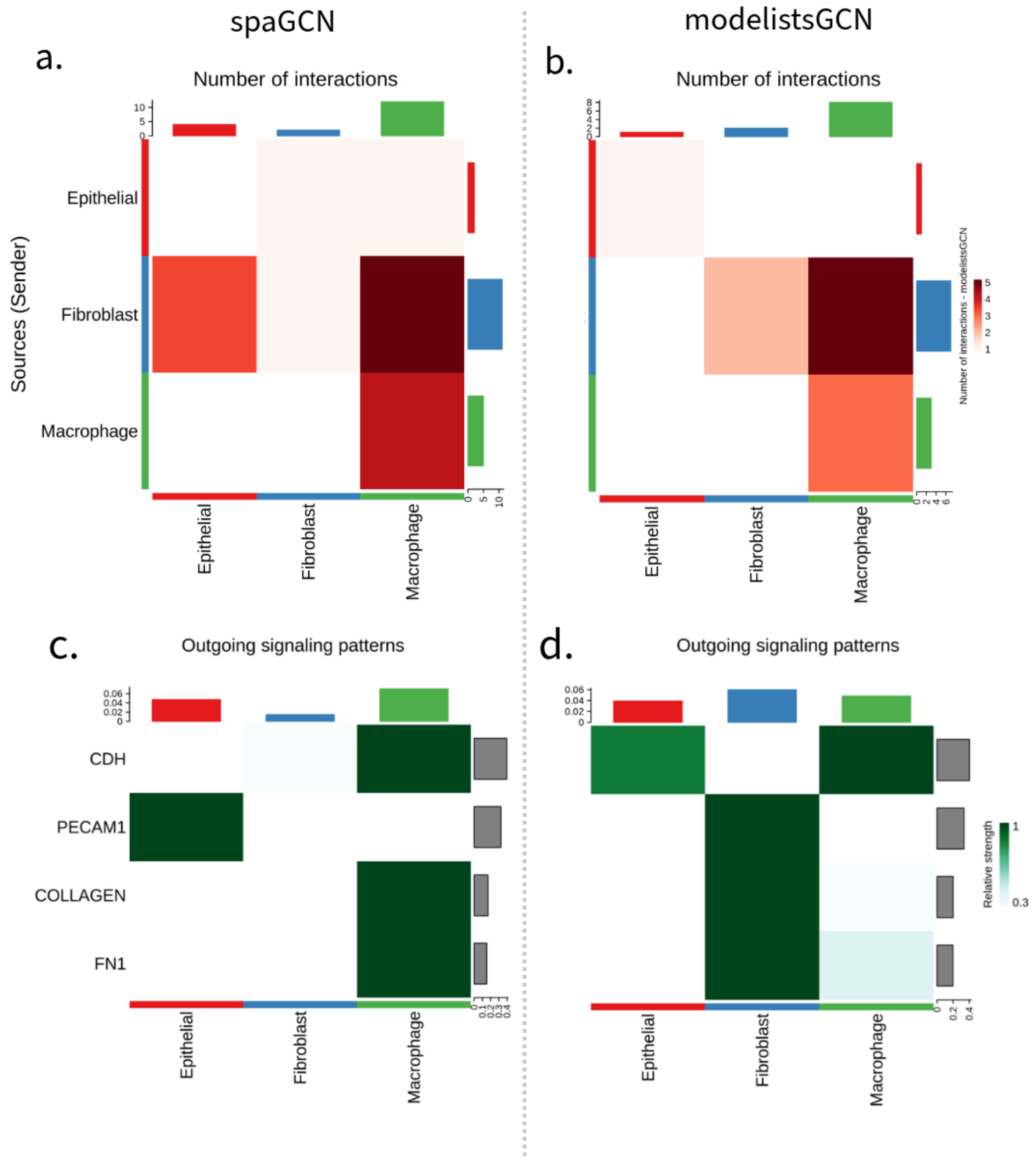

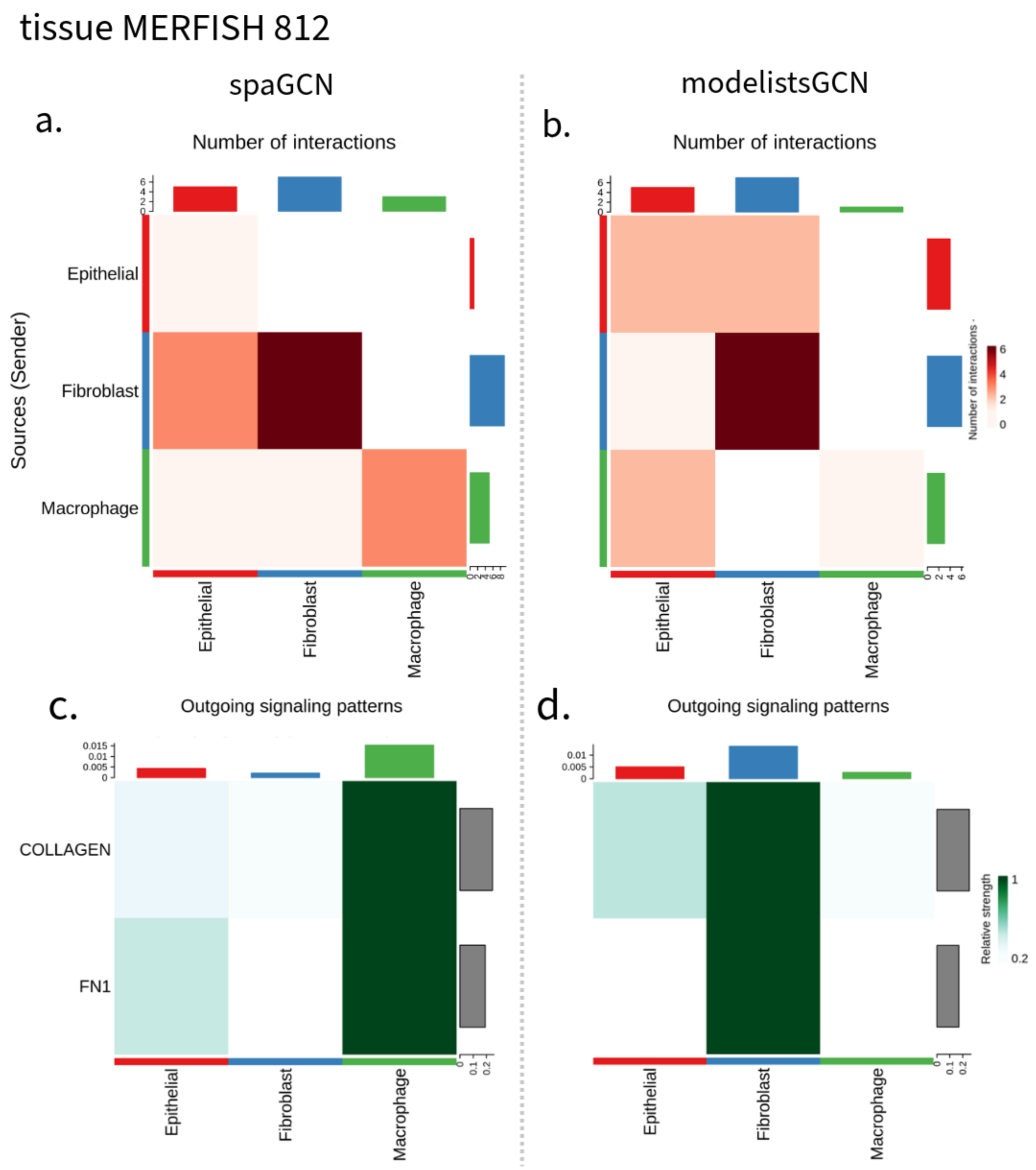

Fig. S12. Cell-cell communication analysis inferred by CellChat in MERFISH breast cancer metastasis tissues. Three tissues are shown (MERFISH 878, 313, and 812), selected at random from the 10 samples analyzed. Only cell types that are commonly detected by both spaGCN and ModelistsGCN are included in the analysis. These analyses follow the CellChat framework[3], which models intercellular communication networks by integrating known ligand-receptor interactions with gene expression data to infer signaling probability, interaction strength, and pathway-level communication patterns. Comparison is shown between spaGCN-based cell-type annotations (left panels: a, c, e) and ModelistsGCN-based annotations (right panels: b, d, f). **(a–b)** Heatmaps showing the number of inferred ligand-receptor interactions between cell types, with rows representing sender cells and columns representing receiver cells. Bar plots summarize total outgoing and incoming interactions per cell type. **(c–d)** Heatmaps of aggregated outgoing signaling patterns grouped into major signaling pathways (e.g., MHC-II, LAMININ). Color intensity

represents relative signaling strength, normalized across pathways. **(e–f)** Dot plots showing communication probabilities for selected ligand-receptor pairs between cell-type pairs for MERFISH 878. These plots are shown only for this sample, as the corresponding plots for MERFISH 313 and 812 were too sparse for meaningful visualization. Dot color encodes communication probability (from low to high), and dot size indicates statistical significance (*p*-value).

Across tissues, ModelistsGCN tends to assign signaling activity in ways that are more consistent with expected cell-type functions. In MERFISH 878, ModelistsGCN assigns MHC-II signaling to Macrophages (c–d), in line with their antigen-presenting role, whereas spaGCN attributes this pathway to Smooth Muscle cells. Similarly, PECAM1-related signaling is assigned away from Epithelial cells by ModelistsGCN (c–d), whereas spaGCN assigns it to Epithelial cells. In MERFISH 313, ModelistsGCN attributes COLLAGEN and FN1 pathways to Fibroblasts (c–d), consistent with their established role in extracellular matrix production, whereas spaGCN assigns these pathways to Macrophages. In MERFISH 812, ModelistsGCN again assigns outgoing signaling predominantly to Fibroblasts (c–d), in agreement with their role as key stromal signaling cells. One exception is observed in MERFISH 812, where spaGCN infers Fibroblasts as the primary senders toward Epithelial cells (a–b), which may better reflect commonly described stromal-to-epithelial signaling interactions. Overall, however, ModelistsGCN more frequently aligns pathway assignments with expected biological roles.

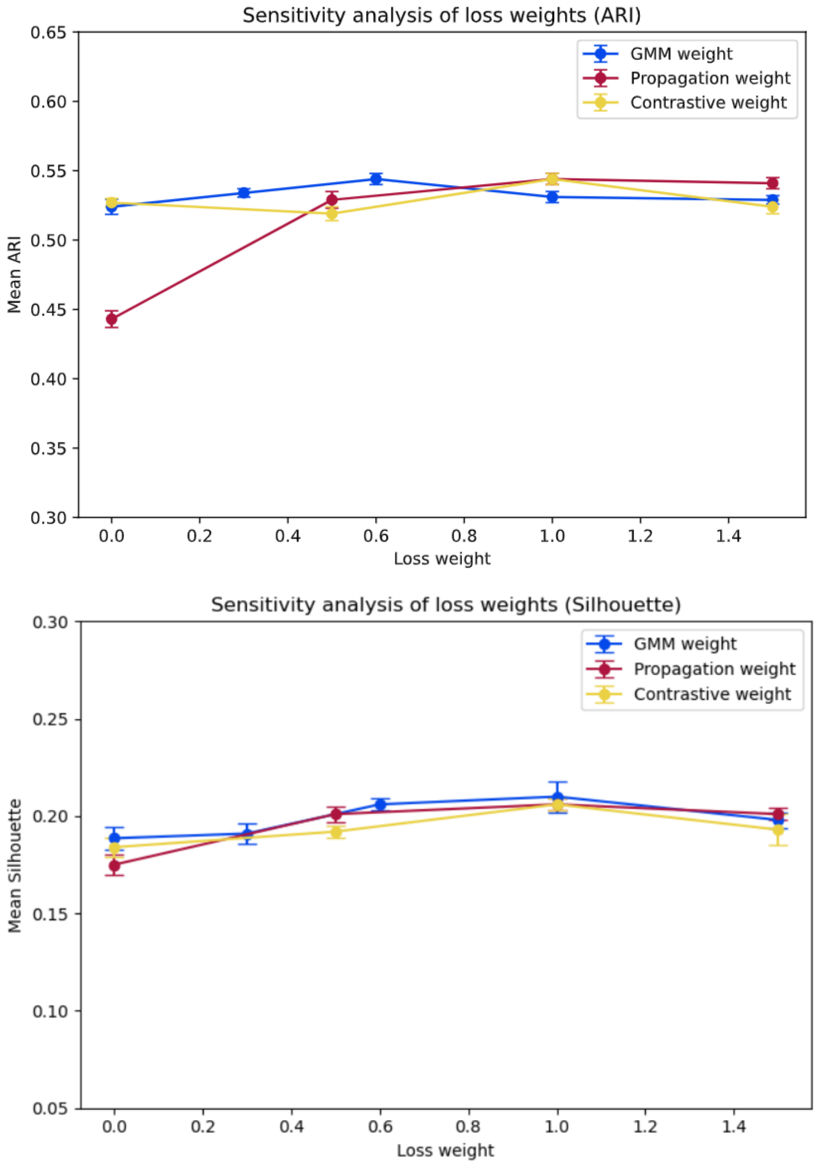

Fig. S13. Sensitivity analysis of loss function weights using the mouse visual cortex ExSeq dataset, for which curated cell-type annotations (ground truth) were available. Mean performance (ARI, top; Silhouette score, bottom) is shown ± standard error across five independent runs with different random seeds. Each curve was generated by varying one loss weight at a time while keeping the others fixed. The default weights are 0.6 for the GMM loss ($\mathcal{L}_{gmm}$) and 1 for the propagation ($\mathcal{L}_{prop}$) and contrastive ($\mathcal{L}_{con}$) losses. Performance remains fairly stable around these default values, with most tested weight changes resulting in variations within ~10% of the corresponding default performance. Larger deviations are observed when setting the propagation loss weight to 0, and to a lesser extent when removing the contrastive loss, highlighting the importance of these components for maintaining clustering performance.

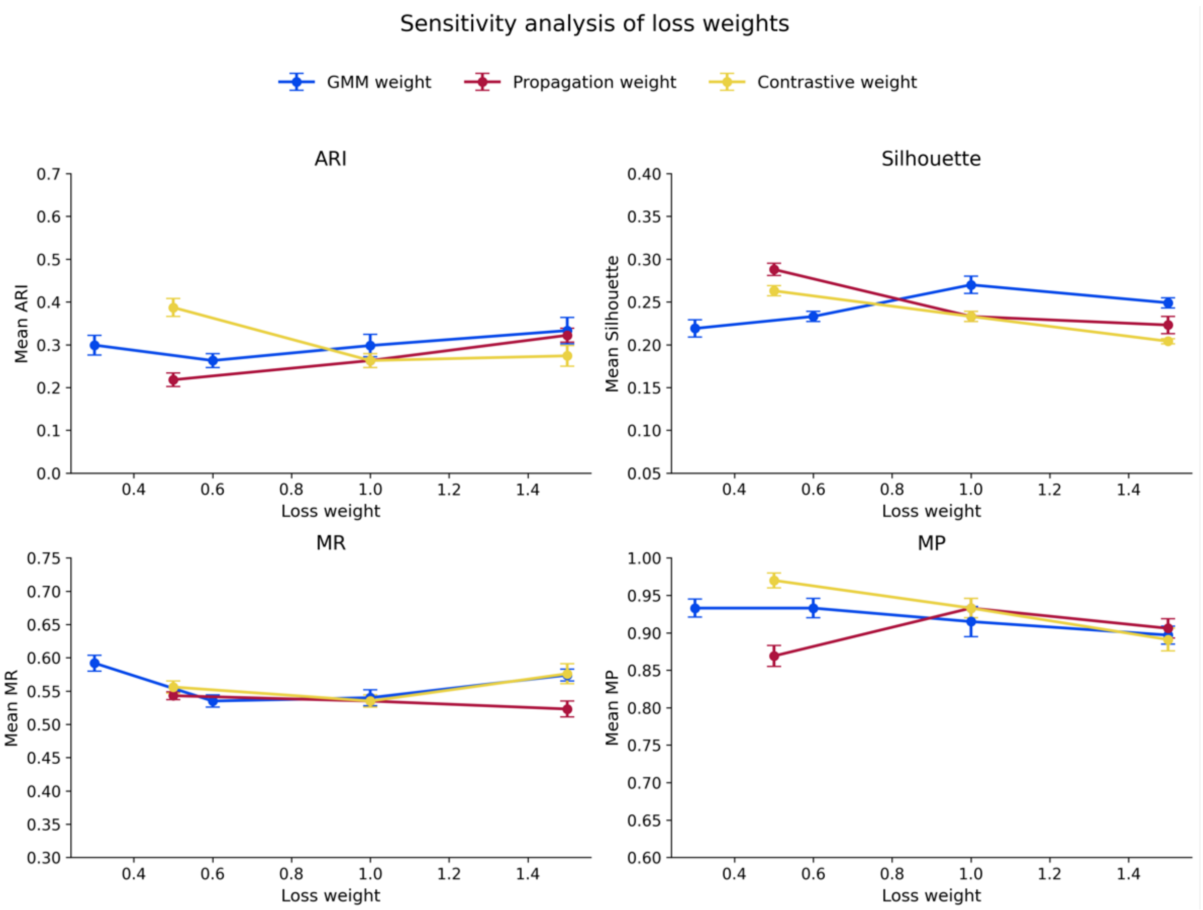

Fig. S14. Sensitivity analysis of loss weights using the MERFISH 313 metastatic breast cancer tissue. Model performance is evaluated across varying weights assigned to the GMM ($\mathcal{L}_{gmm}$), propagation ($\mathcal{L}_{prop}$), and contrastive ($\mathcal{L}_{con}$) loss components. Each curve was generated by varying one loss weight at a time while keeping the others fixed. Each panel reports mean performance (± standard error, STE) across five independent runs with different random seeds for ARI, Silhouette score, marker recall (MR), and marker precision (MP). The tissue was selected at random from the set of ten MERFISH samples analyzed in this study. The default weights are 0.6 for the GMM loss and 1 for the propagation and contrastive losses. Performance remains relatively robust around these default values, with most tested weight changes resulting in variations within ~10% of the corresponding default performance. Larger deviations are observed when substantially reducing the propagation weight and when lowering the contrastive weight, indicating that these components contribute to maintaining both clustering quality and biological consistency.

References

1. Wang H, Huang R, Nelson J, et al. Systematic benchmarking of imaging spatial transcriptomics platforms in FFPE tissues. Nat. Commun. 2025; 16:10215
2. Stringer C, Wang T, Michaelos M, et al. Cellpose: a generalist algorithm for cellular segmentation. Nat. Methods 2021; 18:100–106
3. Jin S, Guerrero-Juarez CF, Zhang L, et al. Inference and analysis of cell-cell communication using CellChat. Nat. Commun. 2021; 12:1088
