# Supplementary material for: ModelistsGCN: a multimodal graph convolutional network framework for single-cell spatial transcriptomic cell typing
Source: Brief Bioinform. 2026 Jun 22;27(3):bbag340. doi: 10.1093/bib/bbag340 (PMC13284712; doi:10.1093/bib/bbag340)
Supplement: Supplementary_Data3_bbag340 [file supplementary_data3_bbag340.pdf]

Supplementary Tables

Method	Scope of work	Primary objective is cell typing	Designed for single cells	Tested on single cells	Tested on low gene panel	Uses spatial position of cells or spots	Uses single cell morphology	Uses H&E or nuclei image
**stMIC** [1]	Deep learning spatial clustering at spot resolution	No	No	No	No	No	No	Yes
**STGNNks** [2]	Spot-level deep learning spatial domain clustering and trajectory analysis	Yes	No	No	No	Yes	No	No
**stLearn** [3]	Spatial graph based trajectory and cell-cell interaction analysis	No	No	Yes (but mostly spot based)	No	Yes	No	Yes

**STELLAR** [4]	Geometric deep learning for spatial cell-type annotation	Yes	Yes	Yes	Yes	Yes	No	No
**GraphST** [5]	Graph neural network spatial clustering and integration	No	No	No	No	Yes	No	No
**SpaGCN** [6]	Graph convolution-based spatial clustering and differential expression	No	No	No	No	Yes	No	Yes
**Bayes Space** [7]	Bayesian spatial clustering	No	No	No	No	Yes	No	No
**SEDR** [8]	Spot-level deep learning spatial clustering and expression denoising	No	No	No	No	Yes	No	No
**CCST** [9]	Single-cell spatial clustering via graph neural networks	Yes	Yes	Yes	No (10,000 genes)	Yes	No	No

**SCAN-IT** [10]	Deep learning spatial domain detection	No	No (spots or cells)	Yes	Yes	Yes	No	Yes
**BASS** [11]	Bayesian single-cell clustering and spatial domain detection	Yes	Yes	Yes	Yes	Yes	No	No
**conST** [12]	Contrastive learning based spot-level spatial learning	No	No (spots or cells)	Yes	Yes	Yes	No	Yes
**Space Flow** [13]	Deep learning spatial embedding and spatio - temporal mapping	No	No	No (only domain analysis with seq-FISH)	Yes	Yes	No	No
**Squidpy** [14]	General purpose spatial omics analysis	No	No	Yes	Yes	Yes	No	Yes
**Modelists GCN**	Single-cell spatial cell typing with graph learning	Yes	Yes	Yes	Yes	Yes	Yes	Yes

Table S1. Comparison of computational methods for spatial cell typing. Comparison of representative spatial transcriptomics analysis methods across key design and evaluation dimensions relevant to single-cell spatial cell typing. For each method, we summarize its scope of work and primary objective, whether it was designed for single-cell data and tested on single-cell-resolved datasets, and whether it was evaluated on low gene panel data

(targeted panels with tens to hundreds of genes, <1,000 genes). We further indicate whether each method incorporates spatial position (cell or spot coordinates), explicit single-cell morphological features, or image-derived information (e.g., H&E or nuclei images, which may be processed using convolutional neural networks or other image-analysis pipelines). Importantly, methods that use histological images do not necessarily model interpretable single-cell morphological properties, as image-based features are often learned implicitly from image representations rather than derived from explicit cell-level measurements. This table was constructed with guidance from the benchmarking study in[15].

Sample ID	Dataset	Cell types with modelist anchors	Cell types discovered without modelist anchors
ExSeq visual cortex	Visual cortex	L6, L6a, L5b, L5, L4, L2/3, Sst	L5/5a, L2
ExSeq 330	Metastatic breast cancer	B cells, Endothelial, Epithelial, Fibroblast	NK
ExSeq 364	Metastatic breast cancer	B cells, Epithelial	T cells, Macrophage, Monocytes, Endothelial
ExSeq 783	Metastatic breast cancer	B cells, Endothelial, Epithelial, T cells	---
ExSeq 880	Metastatic breast cancer	Epithelial, Fibroblast, T cells	---
ExSeq 982	Metastatic breast cancer	,Endothelial, Epithelial Fibroblast, Macrophage	---
MERFISH 313 slice 4	Metastatic breast cancer	Epithelial, Fibroblast	Macrophage
MERFISH 514 slice 9	Metastatic breast cancer	Epithelial, Fibroblast	Macrophage
MERFISH 812 slice 4	Metastatic breast cancer	Epithelial, Fibroblast	Macrophage, Smooth muscle
MERFISH 853 slice 4	Metastatic breast cancer	Epithelial, Fibroblast	Macrophage
MERFISH 878 slice 10	Metastatic breast cancer	,Epithelial, Fibroblast Macrophage	Smooth muscle
MERFISH 880 slice 9	Metastatic breast cancer	Epithelial	Macrophage, Smooth muscle

MERFISH 917 slice 9	Metastatic breast cancer	Epithelial	Macrophage
MERFISH 944 slice 6	Metastatic breast cancer	,Endothelial, Epithelial Macrophage	,Epithelial neuro Fibroblast
MERFISH 982 slice 4	Metastatic breast cancer	Epithelial, Macrophage	Fibroblast, Smooth muscle
MERFISH 982 slice 9	Metastatic breast cancer	Epithelial, Macrophage	Fibroblast

Table S2. Cell types identified with and without prior knowledge. For each sample and dataset, the table summarizes the cell types for which high-confidence modelist anchors were defined based on prior biological knowledge, as well as cell types that were identified by ModelistsGCN without dedicated modelist anchors. The latter represent cell populations that emerged from the data during analysis, without prior specification, highlighting the model's ability to identify additional cell types beyond those explicitly anchored.

Cell type	Marker genes
B cells	IGHG1, IGHG4, IGKC, IGHM, CD79A, TCL1A, BANK1, MS4A1, CD79B
Endothelial cells	RAMP2, PTPRB, PLVAP, SOX18, ADGRL4, CD34, HSPG2, IL3RA, CALCRL, NOTCH1, CD36, PECAM1, GNG11
Tumor cells	,AGR2, FXYD3, KRT19, KRT8, KRT7, ERBB2, S100A14, TSPAN ,CLDN4, CRABP2, EPCAM, SPDEF, SDC1, GRB7, CD24, AZGP1 TFF3, GPR160, KRT18, PGR, CD44, ALDH1A3, CDH1, GATA3
Fibroblast	ACTA2, COL1A1, COL1A2, LUM, DCN, COL3A1, MYL9, TPM2, THY1, MYLK, CSRP2, BGN, SULF1
Macrophage	,FTL, CD68, HLA-DRA, C1QB, C1QA, MSR1, AIF1, CCL3, LYZ ,SPP1, FCGR3A, APOC1, CD74, HLA-DPA1,HLA-DRB1, CD163 APOE, CD14
Monocyte	FCN1, S100A9, S100A8, LGALS2, LST1
T cells	CD3G, CD3E, IL7R, CCL5, CD3D, TRAC, CD2, CD96, CD69, CD7, FOXP3, CD4, CD8A, CD40LG
Hepatocytes	FGFR2, IL32, BICC1, AR
Smooth muscle	FAT1, TAGLN, MMP11
NK	TRDC, NKG7, GNLY, GZMB

Table S3. Marker gene panels used for modelist cell selection in metastatic breast cancer tissues. For each major cell type, we list the marker genes used to define high-confidence modelist cells in the metastatic breast cancer samples analyzed in this study. Markers were derived from differential expression analysis of annotated scRNA-seq reference datasets and complemented with literature-supported canonical markers (see Methods).

Tissue ID	ModelistsGCN	SpaGCN	GraphST	Squidpy
MERFISH 880<br><br>cells 3414	Runtime:10.54s<br>Memory usage:<br>1.298 GB	Runtime: 28.52s<br>Memory usage:<br>1.257 GB	Runtime: 61.54s<br>Memory usage:<br>1.57 GB	Runtime: 22.80s<br>Memory usage:<br>1.416 GB
	Runtime:10.31s<br>Memory usage:<br>1.322 GB	Runtime: 25.53s<br>Memory usage:<br>1.242 GB	Runtime: 62.66s<br>Memory usage:<br>1.567 GB	Runtime: 22.51s<br>Memory usage:<br>1.41 GB
	Runtime: 10.44s<br>Memory usage:<br>1.295 GB	Runtime: 24.11s<br>Memory usage:<br>1.255 GB	Runtime: 61.30s<br>Memory usage:<br>1.92 GB	Runtime: 22.65s<br>Memory usage:<br>1.425 GB
	Runtime: 10.68s<br>Peak RAM: 1.297<br>GB	Runtime: 24.07s<br>Memory usage:<br>1.264 GB	Runtime: 60.95s<br>Memory usage:<br>1.902 GB	Runtime: 22.35s<br>Memory usage:<br>1.425 GB
	Runtime: 10.76s<br>Memory usage:<br>1.316 GB	Runtime: 27.94s<br>Memory usage:<br>1.233 GB	Runtime: 62.84s<br>Memory usage:<br>1.578 GB	Runtime: 23.10s<br>Memory usage:<br>1.425 GB
MERFISH<br>944<br><br>cells 9880	Runtime: 50.41s<br>Memory usage:<br>5.222 GB	Runtime: 82.52s<br>Memory usage:<br>2.538 GB	Runtime: 364.09s<br>Memory usage:<br>5.38 GB	Runtime: 70.49s<br>Memory usage:<br>2.01 GB
	Runtime: 51.13s<br>Memory usage:<br>5.366 GB	Runtime: 74.23s<br>Memory usage:<br>2.615 GB	Runtime: 365.04s<br>Memory usage:<br>5.37 GB	Runtime: 69.83s<br>Memory usage:<br>2.01 GB
	Runtime: 51.50s	Runtime: 68.01s	Runtime: 369.13s	Runtime: 70.08s

	Memory usage: 5.3 GB	Memory usage: 2.609 GB	Memory usage: 5.379 GB	Memory usage: 2.02 GB
	Runtime: 58.31s Memory usage: 5.278 GB	Runtime: 73.29s Memory usage: 2.719 GB	Runtime: 368.68s Memory usage: 5.379 GB	Runtime: 70.15s Memory usage: 2 GB
	Runtime: 55.20s Memory usage: 5.59 GB	Runtime: 87.73s Memory usage: 2.718 GB	Runtime: 370.78s Memory usage: 5.374 GB	Runtime: 70.49s Memory usage: 2.01 GB

Table S4. Runtime and memory usage comparison across spatial clustering methods. Computational performance of ModelistsGCN, SpaGCN[6], GraphST[5], and Squidpy[14] evaluated on two MERFISH datasets: tissue 880 (3,414 cells) and tissue 944 (9,880 cells). Reported values show per-run wall-clock runtime (seconds) and memory usage (GB) across 5 repeated runs under default method parameters. Experiments were conducted on a Linux workstation with a 12th Gen Intel Core i7-12700 CPU, 32 GB RAM.

		Silhouette	ARI	MR	MP
MERFISH - 514	STELLAR	-	-	-	-
	BASS	0.243	0.918	**0.763**	**1**
	CCST	0.220	0.057	0.335	0.866
	ModelistsGCN	**0.265**	**0.940**	0.611	0.806
MERFISH - 878	STELLAR	0.144	0.252	0.418	0.673
	BASS	**0.344**	0.231	0.488	**1**
	CCST	0.208	0.095	0.310	0.8
	ModelistsGCN	0.31	**0.734**	**0.622**	0.786
MERFISH - 313	STELLAR	0.144	0.108	0.507	0.798
	BASS	**0.32**	**0.208**	0.522	0.827
	CCST	0.196	-0.004	0.207	0.714
	ModelistsGCN	0.242	0.195	**0.523**	**0.973**
MERFISH - 812	STELLAR	0.115	0.807	0.27	0.739
	BASS	0.246	0.862	0.402	**1**
	CCST	0.253	0.003	0.109	0.6
	ModelistsGCN	**0.352**	**0.923**	**0.472**	0.737
MERFISH - 853	STELLAR	0.136	-	0.347	0.806
	BASS	0.124	-	**0.417**	0.907
	CCST	0.253	-	0.172	**1**
	ModelistsGCN	**0.372**	**0.671**	0.256	0.625

MERFISH - 880	STELLAR	0.044	0.137	0.312	0.467
	BASS	**0.447**	-0.118	**0.554**	**0.866**
	CCST	0.226	0.139	0.030	0.2
	ModelistsGCN	0.132	**0.899**	0.448	0.661
MERFISH - 917	STELLAR	0.125	0	0.327	0.8
	BASS	**0.252**	0	0.377	**1**
	CCST	0.163	0	0.059	0.8
	ModelistsGCN	**0.252**	**1**	**0.495**	0.818
MERFISH - 944	STELLAR	0.183	0.719	0.392	0.504
	BASS	0.330	0.709	**0.664**	**0.884**
	CCST	0.183	0.120	0.231	0.583
	ModelistsGCN	**0.331**	**0.821**	0.545	0.322
MERFISH - 982 slice 4	STELLAR	0.205	0.386	0.234	0.506
	BASS	0.329	**0.911**	**0.339**	**0.818**
	CCST	0.205	0.128	0.184	0.739
	ModelistsGCN	**0.356**	0.595	0.275	0.588
MERFISH - 982 slice 9	STELLAR	0.229	**0.815**	0.197	0.586
	STELLAR*	0.167	0.236	0.157	0.468
	BASS	**0.343**	0.812	**0.258**	**0.875**
	CCST	0.188	0.087	0.173	0.781
	ModelistsGCN	0.333	0.193	0.245	0.68

Table S5. Benchmarking of ModelistsGCN against STELLAR, CCST, and BASS across MERFISH breast cancer tissues. Clustering performance is reported using Silhouette score, adjusted Rand index (ARI), marker recall (MR), and marker precision (MP) across all tested MERFISH datasets. STELLAR is a reference-based method that relies on spatially annotated cell types from an external reference dataset[4]; accordingly, MERFISH 514 was used as the reference dataset and is not evaluated for STELLAR. For MERFISH 982 (slice 9), an additional comparison is shown using a reference slice from the same tissue (STELLAR*; reference: MERFISH 982 slice 4), to assess whether performance improves when using a more closely matched reference. This was not observed, likely due to spatial heterogeneity between non-adjacent sections (~75 µm apart). Across datasets, ModelistsGCN consistently achieves improved agreement with expected cell types compared to STELLAR, without requiring an external reference dataset, i.e., spatial cell-type annotations from a closely matched tissue, often an adjacent section, which may not be available in practice. Compared to CCST[9], ModelistsGCN shows substantially higher ARI across all comparable datasets (9/9), with an average improvement of ~0.63, along with higher Silhouette scores (9/10 datasets) and consistently improved marker recall (~0.27 on average), while marker precision remains comparable. Compared to BASS[11], ModelistsGCN generally achieves higher ARI (6/9 datasets; average improvement ~0.2-0.25), indicating stronger agreement with expected cell types, while BASS tends to show higher marker precision and ModelistsGCN higher marker recall. Silhouette scores are overall comparable between the methods. In addition to improved or comparable performance, ModelistsGCN provides substantially faster runtimes while maintaining comparable memory usage. For example, MERFISH 880 required ~10 seconds for ModelistsGCN compared to ~3870 seconds for CCST and ~3365 seconds for BASS, and MERFISH 944 required ~54 seconds for ModelistsGCN compared to ~12785 seconds for CCST and ~19220 seconds for BASS. The runtime of STELLAR was comparable to ModelistsGCN. Memory usage for STELLAR and CCST was comparable to ModelistsGCN (Table S4), while BASS showed somewhat higher memory usage, typically within a factor of two. Spatial cell-type annotations for MERFISH 514 and MERFISH 982 slice 4 were obtained from[16]. ARI values for MERFISH 853 were not computed for STELLAR, CCST, and BASS due to uncertainty in cell ID matching.

$\mathcal{L}_{prop}$	X	✓	✓	✓
$\mathcal{L}_{gmm}$	✓	X	✓	✓
$\mathcal{L}_{con}$	✓	✓	X	✓
Silhouette	0.175 ± 0.03	0.189 ± 0.04	0.184 ± 0.02	**0.206 ± 0.02**
ARI	0.443 ± 0.04	0.524 ± 0.03	0.527 ± 0.02	**0.544 ± 0.02**

Table S6. Ablation analysis of loss function components using the mouse visual cortex ExSeq dataset, for which curated cell-type annotations (ground truth) were available. Clustering performance (Silhouette score and ARI) is reported as mean ± standard deviation across five independent runs with different random seeds, reflecting variability due to stochastic model initialization and training. Ablations evaluate the contribution of the propagation loss ($\mathcal{L}_{prop}$), which promotes modelist-guided label propagation across neighboring cells; the GMM pull loss ($\mathcal{L}_{gmm}$), which promotes clustering around Gaussian mixture components; and the contrastive loss ($\mathcal{L}_{con}$), which improves separation in the embedding space. All loss components contribute to performance, with the propagation loss showing the largest effect in the ablation analysis, highlighting the importance of modelist-guided propagation in assigning neighboring cells.

	Silhouette	ARI
Modelists anchoring + Farthest-point GMM initialization	0.206 ± 0.02	**0.544 ± 0.02**
Modelists anchoring + GMM unsupervised	0.21 ± 0.01	0.533 ± 0.03
Modelists anchoring + Random initialization	0.17 ± 0.02	0.54 ± 0.02
GMM unsupervised	**0.24 ± 0.04**	0.39 ± 0.05
Random initialization	0.043 ± 0.04	0.24 ± 0.04

Table S7. Ablation analysis of GMM initialization strategies using the mouse visual cortex ExSeq dataset. Clustering performance (Silhouette score and ARI) on the ground-truth annotated tissue is reported as mean ± standard deviation across five runs with different random seeds, reflecting variability due to stochastic model initialization and training. In the modelist-guided settings ("Modelists anchoring"), known clusters are initialized from modelist anchors. The initialization strategy implemented in ModelistsGCN is Modelists anchoring + Farthest-point GMM initialization. In the Modelists anchoring + GMM unsupervised condition, anchored clusters are preserved, while the remaining clusters are initialized using a standard GMM without farthest-point refinement. In the Modelists anchoring + Random initialization condition, anchored clusters are preserved, while the remaining clusters are initialized by randomly selecting remaining cells as centroids. In the GMM unsupervised condition, all clusters are initialized using a standard GMM without modelist anchoring. In the Random initialization condition, all clusters are initialized by randomly selecting cells as cluster centroids. Results indicate that modelist-guided initialization improves agreement between inferred labels and expected cell types for modelist cells, as quantified by ARI. While unsupervised GMM initialization yields the highest Silhouette score, consistent with its objective of optimizing cluster separation, the reduction in ARI suggests that this increased separation comes at the expense of biologically coherent clusters.

	Silhouette	ARI
ModelistsGCN graph	**0.206 ± 0.02**	**0.544 ± 0.02**
Shuffled spatial location	0.025 ± 0.02	0.239 ± 0.01
Shuffled gene expression	0.012 ± 0.02	0.099 ± 0.02
Shuffled morphology	0.21 ± 0.02	0.537 ± 0.01
ModelistsGCN graph without morphology	0.21 ± 0.04	0.53 ± 0.03

Table S8. Ablation analysis of graph construction using the mouse visual cortex ExSeq dataset, for which curated cell-type annotations (ground truth) were available. Clustering performance (Silhouette score and ARI) is reported as mean ± standard deviation across five independent runs with different random seeds, reflecting variability due to stochastic model initialization and training. Ablations evaluate the contribution of graph components by shuffling spatial locations, gene expression, or morphological features, or by removing morphology from graph construction. For the morphology-shuffling analysis, shuffling was performed after PCA transformation of morphological features, reducing linear dependencies among features prior to permutation. Shuffling spatial locations or gene expression markedly reduces performance, highlighting the importance of spatial organization and molecular information for cell typing. Shuffling or removing morphology also reduced ARI, supporting a contribution of morphological information to biologically coherent clustering in the full ModelistsGCN graph.

	Silhouette	ARI
100% of genes (40 genes)	0.206 ± 0.02	0.544 ± 0.02
80% of genes (32 genes)	0.195 ± 0.04	0.539 ± 0.01
50% of genes (20 genes)	0.163 ± 0.03	0.467 ± 0.05
20% of genes (8 genes)	0.106 ± 0.05	0.367 ± 0.04

Table S9. Sensitivity analysis of reduced gene coverage using the mouse visual cortex ExSeq dataset, for which curated cell-type annotations (ground truth) were available. Clustering performance (Silhouette score and ARI) is reported as mean ± standard deviation across five runs with different random seeds, where in each run genes were randomly subsampled to reduce the full 40-gene panel to 32, 20, and 8 genes, capturing variability due to stochastic model initialization and gene selection. Performance remains largely robust with a 20% reduction in gene coverage, whereas reductions of 50% or more lead to a clear decline in cell typing accuracy and cluster separation, highlighting the dependence of performance on transcriptomic information under more severe gene sparsity.

Cell marker enrichment						
Tissue 514				Tissue 853		
	Cell type	Adjusted *p*-value			Cell type	Adjusted *p*-value
**1**	Paneth Cell Large Intestine Human	9.8×10^{-7}		**1**	Macrophage Lung Human	1.1×10^{-4}
**2**	Microglial Cell Embryonic Prefrontal Cortex Human	3.7×10^{-6}		**2**	Macrophage Pancreas Human	2.8×10^{-4}
**3**	Macrophage Nasopharynx Human	2.8×10^{-5}		**3**	Paneth Cell Large Intestine Human	2.8×10^{-4}
**4**	Myeloid Cell Brain Human	3.1×10^{-5}		**4**	Microglial Cell Entorhinal Cortex Human	2.8×10^{-4}
**5**	Microglial Cell Brain Human	1.4×10^{-4}		**5**	Microglial Cell Superior Frontal Gyrus Human	2.8×10^{-4}
**6**	Macrophage Undefined Human	2.5×10^{-4}		**6**	Macrophage Breast Human	4.2×10^{-4}
**7**	Macrophage Breast Human	4.3×10^{-4}		**7**	Macrophage Intestine Human	4.2×10^{-4}
**8**	Macrophage Intestine Human	4.3×10^{-4}		**8**	Microglial Cell Embryonic Prefrontal Cortex Human	5.7×10^{-4}
**9**	Monocyte Fetal Kidney Human	2.2×10^{-3}		**9**	Dendritic Cell Breast Human	1.2×10^{-3}
**10**	Macrophage Kidney Human	2.4×10^{-3}		**10**	Microglial Cell Brain Human	2.2×10^{-3}

Table S10. Cell-type enrichment analysis for clusters lacking predefined modelist anchors. For further biological validation of cell types lacking predefined modelist anchors, we performed differential expression analysis comparing cells assigned to these types against all other cells. This analysis was conducted on two randomly selected tissues from the MERFISH metastatic breast cancer dataset, MERFISH 514 and MERFISH 853. Differential expression was performed using DESeq2[17] with a Benjamini-Hochberg false discovery rate of 0.1. The resulting gene sets, together with a background consisting of all genes targeted in each tissue, were analyzed for pathway enrichment using Enrichr[18], with functional interpretation based on known cell-type signatures from the CellMarker[19] 2024 database.

The table reports the top 10 enriched CellMarker cell-type signatures for each tissue, ranked by adjusted *p*-value. In MERFISH 514, 5 out of the top 10 enriched terms correspond to macrophage-related signatures, including entries such as “Macrophage Nasopharynx,” “Macrophage Undefined,” “Macrophage Breast,” “Macrophage Intestine,” and “Macrophage

Kidney," with additional related myeloid and monocyte signatures also present. In MERFISH 853, 4 out of the top 10 enriched terms are macrophage-related, and notably the top-ranked term is "Macrophage Lung," with additional macrophage signatures including pancreas, breast, and intestine. Together, these results support the interpretation that the analyzed clusters correspond to macrophage-like cell types, consistent with the assignments made in the original analysis (Table S2).

	Silhouette	ARI
Original 10 μm graph	0.206 ± 0.02	0.544 ± 0.02
5 μm graph	**0.214 ± 0.02**	**0.545 ± 0.02**
15 μm graph	0.185 ± 0.04	0.513 ± 0.02

Table S11. Sensitivity analysis of the spatial neighborhood radius using the mouse visual cortex ExSeq dataset, for which curated cell-type annotations (ground truth) were available. Clustering performance (Silhouette score and ARI) is reported as mean ± standard deviation across five independent runs with different random seeds, reflecting variability due to stochastic model initialization and training. Sensitivity analyses evaluate the effect of varying the spatial proximity threshold used to construct the graph (5 μm, 10 μm, and 15 μm). Clustering performance remains stable across the tested range of spatial radii (5–15 μm), with only minor variations in ARI and Silhouette scores despite substantial differences in edge density (1,548 vs. 2,664 vs. 4,157 bidirectional edges). These results indicate that ModelistsGCN is relatively robust to the choice of spatial neighborhood radius within this range.

	Silhouette	ARI	MR	MP
Original 10 µm graph	0.233 ± 0.07	0.263 ± 0.01	0.535 ± 0.06	0.933 ± 0.08
5 µm graph	0.26 ± 0.06	0.347 ± 0.19	0.566 ± 0.09	0.906 ± 0.08
15 µm graph	**0.277 ± 0.06**	**0.369 ± 0.2**	**0.586 ± 0.1**	**0.94 ± 0.04**

Table S12. Sensitivity analysis of the spatial neighborhood radius using the MERFISH 313 metastatic breast cancer tissue. Clustering performance (Silhouette score, ARI, marker recall, and marker precision) is reported as mean ± standard deviation across five independent runs with different random seeds, reflecting variability due to stochastic model initialization and training on a randomly selected tissue from the ten MERFISH samples. Sensitivity analyses evaluate the effect of varying the spatial proximity threshold used to construct the graph (5 µm, 10 µm, and 15 µm). An increase in performance is observed at 15 µm; however, clustering performance remains comparably high across the tested range of spatial radii (5–15 µm).

References

1. Song J, Lamstein J, Ramaswamy VG, et al. Enhancing spatial transcriptomics analysis by integrating image-aware deep learning methods. Pac. Symp. Biocomput. 2024; 29:450–463
2. Peng L, He X, Peng X, et al. STGNNks: Identifying cell types in spatial transcriptomics data based on graph neural network, denoising auto-encoder, and k-sums clustering. Comput. Biol. Med. 2023; 166:107440
3. Pham D, Tan X, Balderson B, et al. Robust mapping of spatiotemporal trajectories and cell-cell interactions in healthy and diseased tissues. Nat. Commun. 2023; 14:7739
4. Brbić M, Cao K, Hickey JW, et al. Annotation of spatially resolved single-cell data with STELLAR. Nat. Methods 2022; 19:1411–1418
5. Long Y, Ang KS, Li M, et al. Spatially informed clustering, integration, and deconvolution of spatial transcriptomics with GraphST. Nat. Commun. 2023; 14:1155
6. Hu J, Li X, Coleman K, et al. SpaGCN: Integrating gene expression, spatial location and histology to identify spatial domains and spatially variable genes by graph convolutional network. Nat. Methods 2021; 18:1342–1351
7. Zhao E, Stone MR, Ren X, et al. Spatial transcriptomics at subspot resolution with BayesSpace. Nat. Biotechnol. 2021; 39:1375–1384
8. Xu H, Fu H, Long Y, et al. Unsupervised spatially embedded deep representation of spatial transcriptomics. Genome Med. 2024; 16:12
9. Li J, Chen S, Pan X, et al. Cell clustering for spatial transcriptomics data with graph neural networks. Nat. Comput. Sci. 2022; 2:399–408
10. Cang Z, Ning X, Nie A, et al. SCAN-IT: Domain segmentation of spatial transcriptomics images by graph neural network. BMVC 2021; 32:406
11. Li Z, Zhou X. BASS: multi-scale and multi-sample analysis enables accurate cell type clustering and spatial domain detection in spatial transcriptomic studies. Genome Biol. 2022; 23:168
12. Zong Y, Yu T, Wang X, et al. conST: an interpretable multi-modal contrastive learning framework for spatial transcriptomics. bioRxiv 2022; https://doi.org/10.1101/2022.01.14.476408
13. Ren H, Walker BL, Cang Z, et al. Identifying multicellular spatiotemporal organization of cells with SpaceFlow. Nat. Commun. 2022; 13:4076
14. Palla G, Spitzer H, Klein M, et al. Squidpy: a scalable framework for spatial omics analysis. Nat. Methods 2022; 19:171–178
15. Yuan Z, Zhao F, Lin S, et al. Benchmarking spatial clustering methods with spatially resolved transcriptomics data. Nat. Methods 2024; 21:712–722
16. Klughammer J, Abravanel DL, Segerstolpe Å, et al. A multi-modal single-cell and spatial expression map of metastatic breast cancer biopsies across clinicopathological features. Nat. Med. 2024; 30:3236–3249
17. Love MI, Huber W, Anders S. Moderated estimation of fold change and dispersion for RNA-seq data with DESeq2. Genome Biol. 2014; 15:550
18. Chen EY, Tan CM, Kou Y, et al. Enrichr: interactive and collaborative HTML5 gene list enrichment analysis tool. BMC Bioinformatics 2013; 14:128
19. Hu C, Li T, Xu Y, et al. CellMarker 2.0: an updated database of manually curated cell markers in human/mouse and web tools based on scRNA-seq data. Nucleic Acids Res. 2023; 51:D870–D876
